# Differential chromatin accessibility in peripheral blood mononuclear cells underlies COVID-19 disease severity prior to seroconversion

**DOI:** 10.21203/rs.3.rs-1479864/v1

**Published:** 2022-04-07

**Authors:** Nicholas S. Giroux, Shengli Ding, Micah T. McClain, Thomas W. Burke, Elizabeth Petzold, Hong A. Chung, Grecia O. Rivera, Ergang Wang, Rui Xi, Shree Bose, Tomer Rotstein, Bradly P. Nicholson, Tianyi Chen, Ricardo Henao, Gregory D. Sempowski, Thomas N. Denny, Maria Iglesias De Ussel, Lisa L. Satterwhite, Emily R. Ko, Geoffrey S. Ginsburg, Bryan D. Kraft, Ephraim L. Tsalik, Xiling Shen, Christopher Woods

**Affiliations:** Duke University; Duke University; Duke University School of Medicine; Duke University School of Medicine; Duke University School of Medicine; Duke University; Duke University; Duke University; Duke University; Duke University; Duke University; Durham Veterans Affairs Health Care System; Duke University; Duke University School of Medicine; Duke University; Duke University; Duke University School of Medicine; Duke University; Duke University School of Medicine; Duke University School of Medicine; Duke University School of Medicine; Duke University School of Medicine; Duke University; Duke University School of Medicine

## Abstract

SARS-CoV-2 infection triggers profound and variable immune responses in human hosts. Chromatin remodeling has been observed in individuals severely ill or convalescing with COVID-19, but chromatin remodeling early in disease prior to anti-spike protein IgG seroconversion has not been defined. We performed the Assay for Transposase-Accessible Chromatin using sequencing (ATAC-seq) and RNA-seq on peripheral blood mononuclear cells (PBMCs) from outpatients with mild or moderate symptom severity at different stages of clinical illness. Early in the disease course prior to IgG seroconversion, modifications in chromatin accessibility associate with mild or moderate symptoms are already robust and include severity-associated changes in accessibility of genes in interleukin signaling, regulation of cell differentiation and cell morphology. Furthermore, single-cell analyses revealed evolution of the chromatin accessibility landscape and transcription factor motif accessibility for individual PBMC cell types over time. The most extensive remodeling occurred in CD14+ monocytes, where sub-populations with distinct chromatin accessibility profiles were observed prior to seroconversion. Mild symptom severity is marked by upregulation classical antiviral pathways including those regulating IRF1 and IRF7, whereas in moderate disease these classical antiviral signals diminish suggesting dysregulated and less effective responses. Together, these observations offer novel insight into the epigenome of early mild SARS-CoV-2 infection and suggest that detection of chromatin remodeling in early disease may offer promise for a new class of diagnostic tools for COVID-19.

## Introduction

Coronavirus disease 2019 (COVID-19), caused by severe acute respiratory syndrome coronavirus 2 (SARS-CoV-2), manifests with highly variable symptom severity^1^. Infected subjects demonstrate clinical trajectories that range from remaining asymptomatic to developing life-threatening illness. A growing body of evidence suggests that the range of clinical manifestations is the result of different immune responses, and that analysis of chromatin accessibility and gene expression in circulating leukocytes can define underlying molecular mechanisms^2^. Studies have shown suppressed immune responses in subjects with only mild symptoms, as indicated by deficient expression of Type I and III interferons^3^. Subjects with more severe disease demonstrated up-regulation of pro-inflammatory factors, including IL-6 and TNF-alpha^4^. The number of monocytes and associated IL-6, CCL2, and CCL8 production in the peripheral blood also are elevated in subjects with severe COVID-19^5,6^. However, regions of regulatory chromatin that contain transcription factor motifs become accessible prior to downstream gene expression and may offer the potential for even earlier detection of these evolving biological responses^7^.

Analyzing time-dependent changes at the epigenetic level is complicated by the variable time course of COVID-19 across patients, which even in the outpatient setting can range from a few days of asymptomatic viral shedding to prolonged febrile illness. Most studies have used clock time (days since illness onset), a measure that is broadly related to immune response stages in many subjects. Another option is to utilize direct measures of immune maturation to broadly characterize where during the host response to infection a given subject exists at a point in time. The development of specific antibodies (IgG) against SARS-CoV-2 marks an inflection point in a COVID-19 patient’s disease progression, indicating a transition from innate immunity to acquired immunity^8,9^. IgG seroconversion typically occurs within two weeks of symptom onset and roughly coincides with the time that patients without critical illness will see clinical improvement^10,11^. However, limited data are available examining the peripheral blood immune responses early in disease progression and prior to seroconversion. To test the hypothesis that the landscape of chromatin accessibility contains biomarkers that define early molecular mechanisms that underpin divergent immunologic responses in SARS-CoV-2 infection, we performed bulk and single-cell Assay for Transposase-Accessible Chromatin using sequencing (ATAC-seq) and RNA-seq on longitudinal PBMC samples from patients before and after seroconversion.

## Results

### A clinical cohort to study early COVID-19 with mild or moderate symptoms

To establish a cohort for the longitudinal study of early SARS-CoV-2 infection, subjects were selected from a large ongoing community-based prospective cohort, the Molecular Epidemiological Study of Suspected Infection (MESSI) in four experimental groups: 1) pre-pandemic healthy controls, 2) subjects exposed to SARS-CoV-2 as close contacts but negative for SARS-CoV-2 by qPCR and serology, 3) outpatients positive for SARS-CoV-2 by qPCR whose maximal illness was mild and 4) outpatients positive for SARS-CoV-2 by qPCR whose maximal illness was moderate. Demographic characteristics of study participants are shown in Table 1. Symptoms associated with COVID-19 illness were self-reported to clinic personnel using a COVID-19 symptom survey developed by Duke University Medical Center consisting of 38 symptoms. Each subject reported a symptom and perceived severity of that symptom from 0 (none) to 4 (very severe). Subjects with mild symptoms exhibited a mean score of 12.9 ± 2.1 and 12.2 ± 2.8 for total PBMC assays (bulk) and assays of individual PBMCs (single-cell assays), respectively, which corresponded to the World Health Organization (WHO) Ordinal 8-point scale 1 (OS1, ambulatory and with no limitation of activities); subjects with moderate symptoms exhibited a mean score of 33.6 ± 2.4 and 36.0 ± 2.3 respectively which corresponded to the WHO Ordinal 8-point scale 2, (OS2, ambulatory and with limitation of activities). Interestingly, despite lacking any evidence of infection (negative SARS-CoV-2 qPCR and serological testing for at least two months after exposure) close household contacts who were exposed to SARS-CoV-2 but remained negative for infection showed broadly similar quantitative symptom scores to subjects with mild disease, with a mean score of 9.9 ± 2.9 and 3.3 ± 2.8 respectively (Table 1). The sum of symptom severity across all symptoms reported was able to distinguish moderate subjects from mild or close contacts but did not distinguish mild from close contacts (Fig. S1A–B). However, the specific symptoms experienced, and their severity, were different between all three groups (Fig. S1C–D). Loss of taste and smell, headache, malaise, and fatigue were most common in the moderate group. Coughing was most common in the mild group and runny nose in the close contacts.

### Transcriptional profiles of PBMCs distinguish mild or moderate COVID-19 from healthy controls

**Figure 1:**
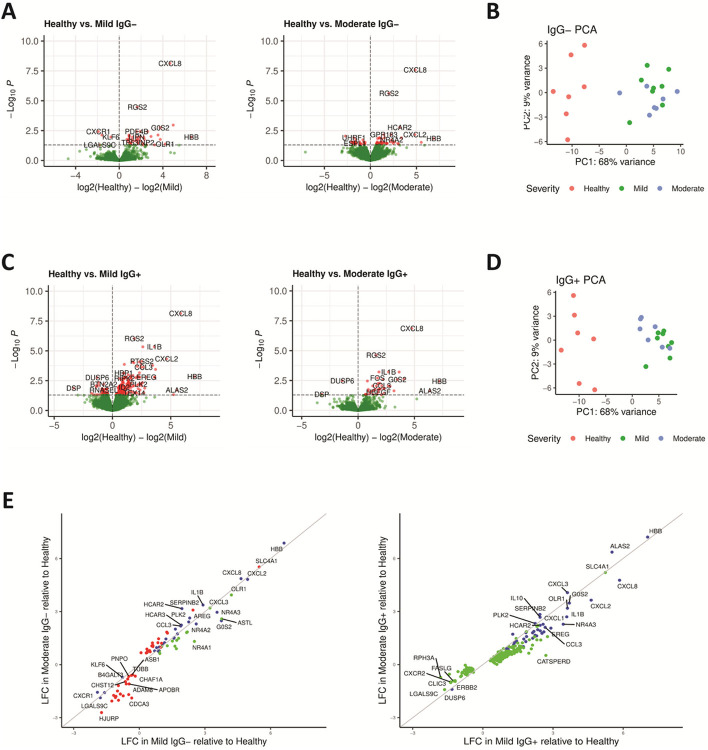
Differential gene expression in peripheral blood mononuclear cells (PBMCs) distinguishes COVID-19 symptom severity. **(A)** Volcano plot depictions of differential gene expression from comparisons of healthy controls to IgG− subjects with mild symptoms (left), healthy controls to IgG− subjects with moderate symptoms (center) or IgG− subjects with mild symptoms compared to subjects with moderate symptoms (right). Differential expression in genes with p <= 0.05 are plotted in red. **(B)** Principal component analysis (PCA) using differentially expressed genes identified in IgG− subjects with mild symptoms (green) and moderate subjects (blue) show distinct separation from healthy controls (red). **(C)** Volcano plot depictions of differential gene expression from comparisons of healthy controls to IgG+ subjects with mild symptoms (left), healthy controls to IgG+ subjects with moderate symptoms (center), or IgG+ subjects with mild symptoms compared to subjects with moderate symptoms (right). Genes with p <= 0.05 are plotted in red. **(D)** PCA using differentially expressed genes identified in IgG+ subjects with mild symptoms (green) and moderate subjects (blue) show distinct separation from healthy controls (red). **(E)** Log fold change (LFC) in differentially expressed genes in IgG− subjects with mild symptoms as a function of IgG− subjects with moderate symptoms (left) and likewise in IgG+ subjects (right). Genes differentially expressed in mild compared to healthy are green, moderate compared to healthy are red and genes differentially expressed in mild or moderate compared to healthy controls are blue.

To identify underlying molecular mechanisms of disease severity in mild or moderate COVID-19 both prior and after seroconversion, PBMCs were isolated and profiled as total PBMCs (bulk assays) or as single nuclei isolated from individual PBMCs (single cell assays). Longitudinal sample collection from each subject spanned the transition from seronegative (IgG−) to seropositive (IgG+) for SARS-CoV-2 spike protein in the mild and moderate symptom groups (Supplementary Table S1 and S2). Differential gene expression analysis of bulk RNA-seq data from mild IgG− COVID-19 subjects compared to healthy controls identified a transcriptional signature of 45 genes with p < = 0.05; moderate IgG− COVID-19 subjects compared to healthy controls identified 71 genes with p < = 0.05 (Fig. 1A). Similar comparisons using RNA-seq from mild IgG + subjects compared to healthy controls or moderate IgG + subjects compared to healthy controls identified 250 and 43 genes, respectively, with p < = 0.05 (Fig. 1C). The gene symbols, gene names, fold change and p values adjusted for multiple hypothesis testing for the four comparisons are found in Supplemental Table S3. Using principal component analysis (PCA), differential gene expression between healthy controls and subjects with either mild symptoms (Fig. 1B) or moderate symptoms (Fig. 1D) were sufficient to distinguish the groups. However, both mild and moderate subjects clustered together and could not be easily distinguished. Considering the level of gene expression (log fold change, LFC) in mild and moderate subjects compared to healthy controls, differentially expressed genes were identified in only mild, only moderate or both symptom cohorts (Fig. 1E). Comparing IgG-subjects, we identified 30 genes observed in both mild and moderate cohorts. In contrast, we did not identify any genes solely associated with moderate IgG + subjects and identified a further 43 genes observed in both IgG + mild and moderate cohorts. Healthy controls exhibited higher expression of the interleukin-8 precursor CXCL8 compared to other groups. Conversely, the interleukin-8 receptor, CXCR1, had higher expression in mild and moderate IgG− subjects. Lower expression of chemokine CCL3 and cytokine IL1B^12^, and higher expression of the myeloid cell plasticity regulator KLF6^13^ was observed in both IgG− and IgG + COVID-19 subjects.

### Chromatin remodeling prior to seroconversion can distinguish mild from moderate COVID-19

**Figure 2:**
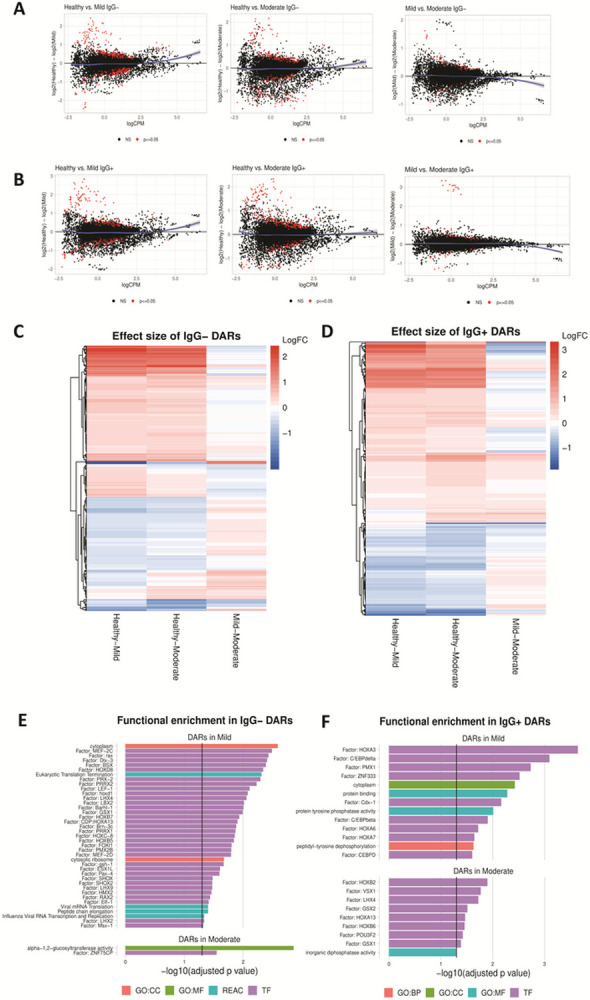
Remodeling of the chromatin landscape identifies gene regulatory markers associated with symptom severity. **(A)** Differential chromatin accessibility, comparing healthy controls to mild symptom IgGsubjects (left) and healthy to moderate symptom IgG− subjects (center), and IgG− mild to moderate symptoms (right). Regions that are significantly different in each comparison at p<= 0.05 are red; regions not significantly different are black. A loess curve fit to the results is plotted in blue. **(B)** Differential chromatin accessibility, comparing healthy controls to mild symptom IgG+ subjects (left), healthy controls to moderate symptom IgG+ subjects (center), and mild to moderate IgG+ subjects (right). A loess curve fit to the results is plotted in blue. **(C-D)** Heat map depiction of differentially accessible regions (DARs) that compares healthy to mild symptoms (left column), healthy to moderate symptoms (center column) and mild to moderate symptoms (right column) in IgG− subjects (C) and in IgG+ (D). **(E-F)** Functional enrichment analysis of DARs in the TRANSFAC database (purple), the REACTOME database (red), and gene ontologies (GO) in categories of biological processes (BP, orange), cellular component (CC, blue), and molecular function (MF, green). DARs identified comparing healthy to mild symptoms (top) or healthy compared to moderate symptoms (bottom) are shown for IgG− **(E)** and IgG+ **(F)** subjects.

To test whether chromatin accessibility can distinguish COVID-19 disease severity in individuals early in their disease course (prior to seroconversion) an Assay for Transposase-Accessible Chromatin using sequencing (ATAC-seq)^14^ was used to quantify differential chromatin accessibility in three groups: subjects with mild symptoms, subjects with moderate symptoms and pre-pandemic healthy controls. A set of 455 differentially accessible chromatin regions (DARs) was found to distinguish IgG− subjects with mild symptoms from those with moderate symptoms at p < = 0.05 (Fig. 2A). Of these markers, 73 regions shared differential accessibility in mild and moderate IgG− subjects, demonstrating both severity-associated evidence of chromatin remodeling and a smaller conserved epigenetic response. Additionally, a set of 375 regions of differentially accessible chromatin was identified in IgG + subjects (Fig. 2B). DARs specific to the comparisons in 2A-B as heat map depictions (Fig. 2C–D) for prior and post seroconversion shows overlap between mild and moderate symptom subjects compared to healthy controls. Functional enrichment was then performed for each set of differentially accessible regions to identify an association with transcription factor motifs and downstream pathways. In IgG− subjects with mild symptoms, we found an enrichment of transcription factors and pathways related to viral infection (Fig. 2E). Fewer enriched pathways were observed in the IgG− subjects with moderate symptoms. Transcription factors found to be enriched in accessible chromatin from IgG + subjects with mild symptoms were associated with dysregulation of myeloid development via HOXA3, C/EBP-B and C/EBP-D^15^ (Fig. 2F).

### CD14 + monocytes are the primary cell type differentially activated in IgG− subjects

**Figure 3:**
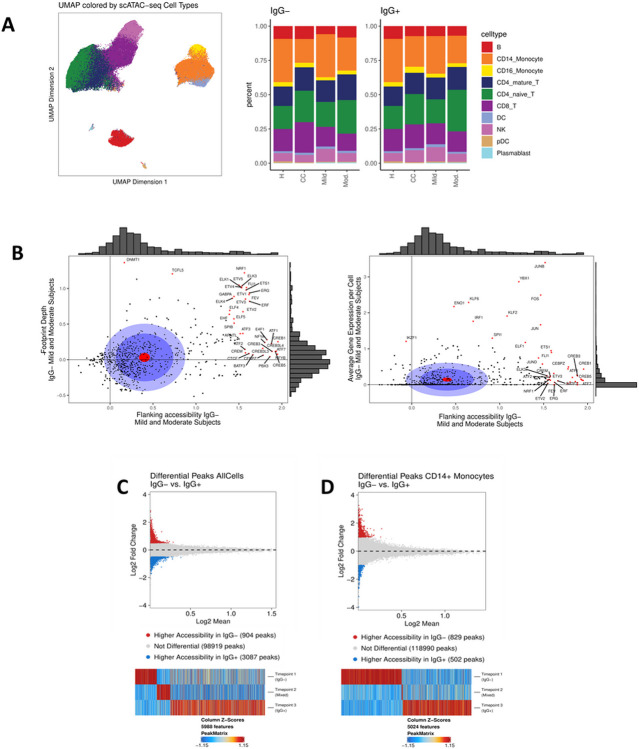
Epigenetic signatures in cells collected from COVID-19 subjects evolve with disease progression. **(A)** Uniform manifold approximation and projection (UMAP) plot of single-cell ATAC-seq data generated from healthy controls, uninfected close contacts, and COVID-19 subjects with mild or moderate symptoms (left). Relative abundance of cell types represented in the scATAC-seq data are plotted for each group at IgG− and IgG+ timepoints for COVID-19 subjects and collection day 0 and 14 for close contacts (right). **(B)** Transcription factor motif accessibility and binding activity measured by footprint depth (left) or average per-cell gene expression (right) in COVID-19 subjects with mild or moderate symptoms. Distribution of accessibility and gene expression are plotted as histograms along the axes (gray). The red circle is the average of all points; the dark blue circle is 50% of all data; the light blue circle is 75% of all data. Motifs with the top 5% change in flanking accessibility are plotted in red. **(C)** Differentially accessible chromatin between IgG− and IgG+ timepoints in all cells and **(D)** CD14+ monocytes. Differentially accessible chromatin observed across the IgG−, mixed and IgG+ timepoints in COVID-19 subjects is shown where significance is defined as p <= 0.05 and absolute LFC > = 0.5 (top); Bottom: Changes in differentially accessible chromatin are shown across time (bottom).

The chromatin accessibility signature detected in the bulk ATAC-seq datasets identified candidate epigenetic biomarkers associated with COVID-19 severity and showed that PBMCs undergo extensive chromatin remodeling in response to SARS-CoV-2 infection. To understand how each cell type contributed to this signature and track the evolution of the chromatin landscape of each cell type during seroconversion, we performed single-cell (sc)ATAC-seq profiling of the PBMCs isolated from subjects with mild or moderate symptoms and healthy controls (Table 1 and Supplemental Table 2). The relative abundance of cell types collected from each subject cohort were similar between IgG− and IgG + timepoints (Fig. 3A). Analysis of transcription factor motif accessibility was applied to PBMCs from IgG− mild or moderate subjects to identify shared regulatory mechanisms. Transcription factors with highly accessible motifs and low gene expression were identified, consistent with epigenetically primed chromatin remodeling without corresponding transcriptional activity (Fig. 3B). These motifs, including those of the CREB and ETS protein families, are characterized by increased binding activity as measured by footprint depth and relatively low average per-cell gene expression. Inflammatory regulators including JUN/FOS, proteins from the KLF family, and IRF1 showed elevated gene expression. A complete list of transcription factors associated with accessible regions and low gene expression are found in Supplemental Table S4.

Comparison of PBMCs collected from subjects with mild or moderate symptoms identified specific peaks enriched on the day of study enrollment (IgG−) versus a timepoint 2–4 weeks later (IgG+) for all cell types (Fig. 3C). A total of 5988 peaks were identified as uniquely accessible at one of the three longitudinal (IgG−, mixed serology, and IgG+) timepoints. A similar experimental approach was applied to each major cell type individually to determine the extent of chromatin remodeling prior to seroconversion. The cells with enrichment of significant peaks at the early, IgG− timepoint were primarily CD14 + monocytes (Fig. 3D).

### Domains of regulatory chromatin are specially enriched in myeloid cells

**Figure 4:**
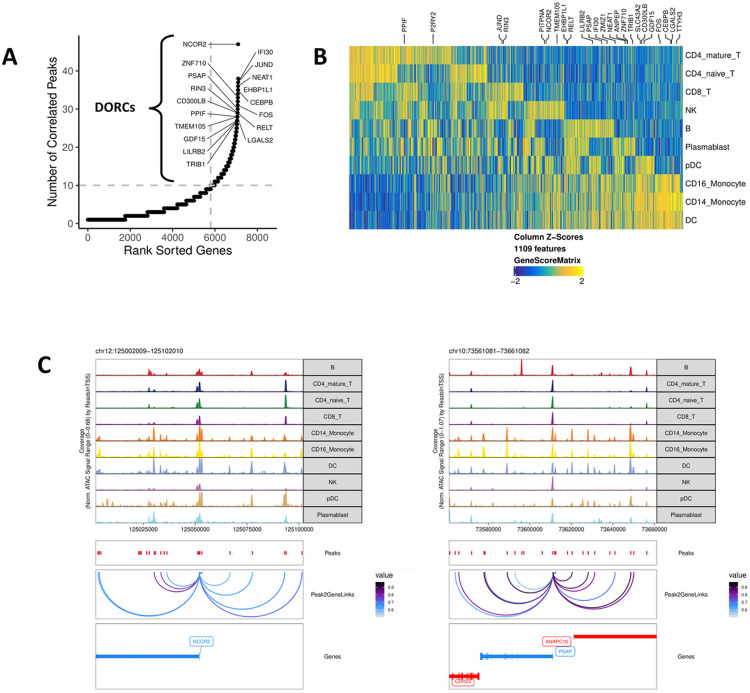
Domains of regulatory chromatin (DORCs) are enriched in myeloid cells, indicating epigenetic control of cell fate. **(A)** Number of peak-to-gene linkages in cells from IgG− subjects is shown by a plot of rank sorted genes as a function of number of correlated peaks. Genes with > 10 linkages are defined as DORCs. Labeled genes are known to be regulated by a super-enhancer. **(B)** DORC gene activity for 1109 loci plotted for each cell type (right). Labeled DORC genes are known to be regulated by a super-enhancer (top). **(C)** Super-enhancer regulated DORC genes for nuclear receptor corepressor 2 (NCOR2, left) and prosaposin (PSAP, right). Peak-to-gene linkages in CD14+ monocytes and other myeloid cells are plotted with a correlation cutoff of 0.5.

Domains of regulatory chromatin (DORCs) were identified in all cell types collected from IgG− subjects with mild or moderate symptoms by counting the number of peak-to-gene linkages for each gene in the scATAC-seq datasets^7^. A total of 1109 genes with greater than 10 peak-to-gene linkages were defined as DORC genes, and those known to be regulated by a super-enhancer were labeled (Fig. 4A). These DORCs showed cell type-specific profiles and appeared to have higher accessibility than gene expression in these cells, suggesting that these genes are being primed for activation (Fig. 4B). Accessibility at the DORCs regulated by a super-enhancer was enriched in CD14 + monocytes and other myeloid cells, including dendritic cells and CD16 + monocytes, indicated by the presence of proximal gene regulatory elements with correlated accessibility to DORC gene loci (Fig., 4C). These DORC genes regulated by super-enhancers play a role in priming active chromatin states in CD14 + monocytes, consistent with the cell fate decisions identified that distinguished subjects with variable symptom severity.

### Epigenetic profiles of CD14 + monocyte subpopulations differentiate mild from moderate IgG− COVID-19

**Figure 5:**
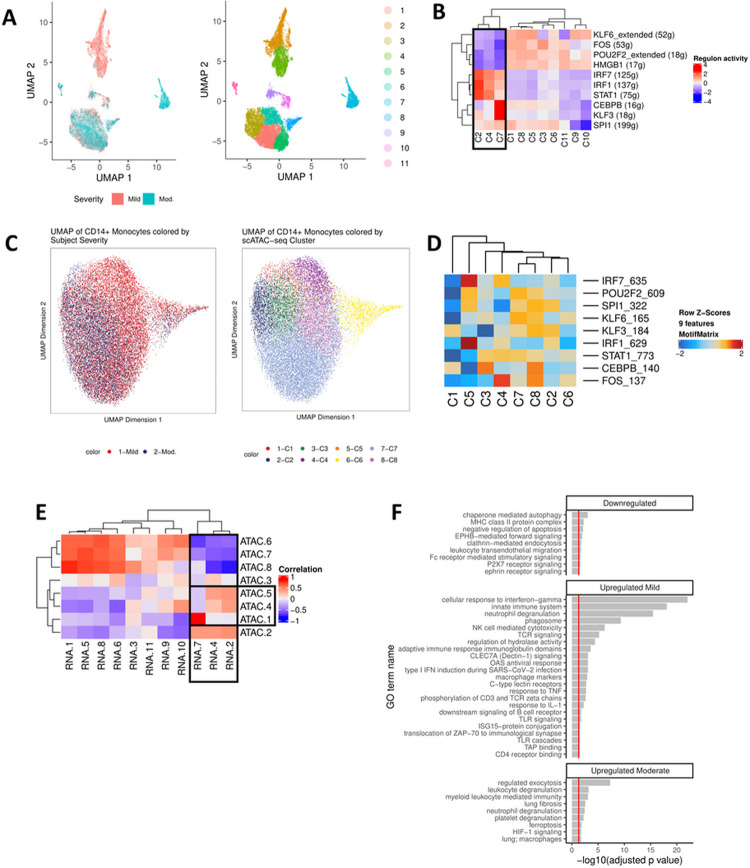
CD14+ monocytes undergo extensive chromatin remodeling prior to seroconversion and harbor severity-specific epigenetic biomarkers. **(A)** scRNA-seq UMAP of all CD14+ monocytes collected from mild and moderate IgG− subjects. UMAP colored by disease severity (left) and cluster number (right). **(B)** Heat map depiction of regulon activity computed for each scRNA-seq cluster using SCENIC. Activity of the top 10 regulons (right) is plotted for each cluster of cells from the UMAP (bottom). The black box indicates clusters of interest. **(C)** scATAC-seq UMAP of all CD14+ monocytes collected from IgG− mild and moderate subjects. UMAP colored by disease severity (left) and cluster number (right). **(D)** Heat map depiction of transcription factor motif enrichment of identified regulons (right) plotted for each scATAC-seq cluster (bottom). **(E)** Correlation plot using regulon activity to link clusters between scRNA-seq (columns) and scATAC-seq (rows). Black box indicates clusters of interest. **(F)** Functional enrichment analysis for regulons down-regulated (KLF6, FOS, POU2F2, HMGB1) or up-regulated (mild: IRF7, IRF1, STAT1; moderate: CEBPB, KLF3) in scRNA-seq clusters 2, 4, and 7 (panel E).

We next identified transcriptomic and epigenetic profiles of sub-populations of CD14 + monocytes that distinguished IgG− subjects with mild from moderate symptoms. Single-cell gene expression data for CD14 + monocytes were processed using the uniform manifold and projection (UMAP) algorithm. Clusters 2 and 4 identified a unique sub-population of CD14 + monocytes in subjects with mild symptoms and cluster 7 identified a sub-population of CD14 + monocytes in subjects with moderate symptoms (Fig. 5A). Transcription factors and downstream targets were identified for each cluster. CD14 + monocyte subpopulations from subjects with mild symptoms exhibited significant up-regulation of the regions that regulate IRF7, IRF1, and STAT1 transcription factors, whereas the CD14 + monocyte subpopulations from subjects with moderate symptoms were characterized by up-regulation of the CEBPB and KLF3 regulatory regions (Fig. 5B).

Single-cell ATAC-seq data for CD14 + monocytes were processed using a similar approach to identify clusters with correlated gene expression regulatory activity (Fig. 5C). Activity at the transcription factor motifs was estimated, and the correlations between scRNA-seq and scATAC-seq clusters were calculated in a pairwise manner (Fig. 5D). Differential gene expression between the mild and moderate subpopulations identified interferon-stimulated gene activation as a distinguishing feature of symptom severity. The cell clusters from scATAC-seq with high correlations with clusters 2, 4, and 7 from scRNA-seq were highlighted. We observed that scATAC-seq clusters 4 and 5 were correlated with scRNA-seq clusters 2 and 4, which identified similar populations of CD14 + monocytes from mild subjects (Fig. 5E). We also observed that scATAC-seq cluster 5 was highly correlated with scRNA-seq cluster 7, identifying similar populations of CD14 + monocytes from moderate subjects. In addition to these transcriptomic markers, functional enrichment analysis was then performed for the transcription factors with differential activity and their downstream target genes (Fig. 5F). Pathways identified centered on those of the adaptive immune response. Differential regulation of pathways related to the transition from innate to adaptive immunity, marked by dectin-1 and the toll-like receptor cascade, suggested that activation of T and B cell receptor signaling were observed in these sub-populations^16–18^.

## Discussion

This study demonstrates that the epigenomes of critical subsets of PBMCs are remodeled extensively early in SARS-CoV-2 infection and reflect disease severity in IgG− subjects with mild and moderate symptoms. Specifically, differential activity of transcription factors and chromatin accessibility prior to anti-spike IgG seroconversion were observed to distinguish disease severity, preceding the later transcriptional response. The fact that these signals are robust even in a small cohort of subjects with subtle differences in disease severity (WHO OS 1 vs 2) suggests the potential power of epigenetic approaches to classify subsets of SARS-CoV-2 infection. This study complements previous work that identified signatures of accessible chromatin in severe or convalescing COVID-19^19–22^

The data show that transcription factor motifs enriched in peaks accessible prior to seroconversion had the highest occupancy in cells from the myeloid lineage, specifically CD14+ monocytes, dendritic cells, and CD16+ monocytes. KLF and CREB transcription factor families, which are known to regulate monocyte-macrophage polarization, have elevated motif accessibility in subjects with mild or moderate symptoms, suggesting an epigenetic priming mechanism controlling cell fate^23,24^. This distinction is important as not only are CD14+ monocytes generally more activated in early COVID-19, as has been described, but here they are shown to differentially activated across subjects with variable disease severity. These differences include modulation of IFN-γ, as reported previously. Specifically, mild symptom severity (or effective control) is marked by upregulation of classical antiviral pathways including those regulating IRF1 and IRF7. With increased severity, these antiviral signals diminish, suggesting dysregulated and less effective responses underlie moderate disease. These early epigenetic changes occur prior to transcriptional manifestations and in this cohort were also more stable and predictive of disease severity than gene expression alone. These findings suggest that epigenetic approaches focusing on chromatin accessibility may offer even greater promise than other host-based molecular diagnostics and predictive tools, as has been suggested for DNA methylation-based profiles^25^. Our work is consistent with previous studies that identified DNA-methylation signatures, which included viral response and interferon signaling, that will predict SARS-CoV-2 infection and clinical outcome^25^ and with genome-wide DNA methylation signatures associated with severe COVID-19 that highlight hypermethylation in IFN-related genes, hypomethylation in inflammatory genes and increased epigenetic age^26^.

Interestingly, CD14+ monocytes underwent the most extensive chromatin remodeling over time and exhibited epigenetic profiles at early seronegative times that distinguish disease severity. In contrast, after seroconversion motifs enriched in accessible peaks were not seen in monocytes, instead showing the highest activity in B cells and plasmablasts, consistent with the transition from inflammatory signaling to adaptive immune development in this phase. Our work is consistent with epigenetic profiles of immune cells of individuals convalescing from COVID-19 that show establishment of immunological memory^22^.

There are limitations to this study, including the relatively small sample size, nonstandard enrollment and sampling times that vary across individuals, and a lack of subjects with critical illness to examine how these epigenetic changes manifest in more severe disease. However, development of a host-response assay that leverages the highly sensitive epigenetic biomarkers established early during infection has the potential to fill a clear unmet clinical need in the care of patients with COVID-19^27,28^. In summary, the evolution of the chromatin landscape in circulating leukocytes during COVID-19 primes host immunological responses at early times, is mediated primarily by CD14+ monocytes and correlates with an observed divergence in disease severity. These changes temporally precede transcriptional manifestations of pathways related to adaptive immunity. Together, these observations offer novel insight into severity-associated variations in host responses to SARS-CoV-2 infection and suggest that detection of critical components of chromatin remodeling in early disease may offer promise for a new class of diagnostic tools for COVID-19.

## Methods

### Cohort recruitment, biological sample collection, and initial phenotyping

Protection of human subjects was in accordance with research protocols approved by the Duke University Institutional Review Board, consistent with the Declaration of Helsinki. Written informed consent was obtained from all research subjects or their legally authorized representatives. Subjects with confirmed or suspected SARS-CoV-2 infection or their close contacts were identified in the outpatient setting and enrolled into the Molecular and Epidemiological Study of Suspected Infection protocol (MESSI, IRB Pro00100241). All close contacts and subjects with mild or moderate COVID-19 were longitudinally sampled from enrollment to convalescent phase. Biological samples and demographic information were collected prospectively at first visit (Day 0) and at weekly intervals on Day 7 and Day 14. At each visit, infection with SARS-CoV-2 was confirmed using quantitative polymerase chain reaction (qPCR) of nasopharyngeal (NP) swab samples, and serology testing was performed for IgG against the SARS-CoV-2 spike domain. All subjects with mild or moderate COVID-19 progressed from seronegative (IgG−) to seropositive (IgG+). Close contacts were qPCR negative and IgG− at all time points; healthy controls were enrolled pre-pandemic and were not tested for SARS-CoV-2 or spike protein IgG. Self-reported symptom surveys were performed at each visit. To quantify symptom severity, the sum of 38 defined symptoms, each scored 0–4 (0-none, 1-mild, 2-moderate, 3-severe, 4-very severe), was determined from symptom onset through each longitudinal sample collection. SARS-CoV-2 q-PCR tests used virus RNA extracted from NP samples in 140 uL of viral transport medium (VTM) according to manufacturer’s instructions (QiaAmp Viral RNA minikit). SARS-CoV-2 nucleocapsid (N1) and human RNase P (RPP30) RNA copies were determined using 5 μL of isolated RNA in the CDC-designed kit (CDC-006–00019, Revision: 03, Integrated DNA Technologies 2019-nCoV kit). SARS-CoV-2 IgG ELISA tests for antibody response were performed using the anti-SARS-CoV-2 spike S1 domain IgG ELISA assay (EUROIMMUN Medizinische Labordiagnostika AG, Lübeck, Germany).

### Purification of PBMCs

PBMCs were purified using the Ficoll-Hypaque density gradient method according to manufacturer’s instructions. Briefly, whole blood was collected in ACD Vacutainer tubes (BD) and processed within 8 hours by dilution 1:2 in PBS, layered onto the Ficoll-Hypaque (Sigma Aldrich) in 50 ml conical tubes, and centrifuged at 420 × g for 25 minutes. Buffy coat was collected, washed twice in D-PBS by centrifugation at 400 × g for 10 minutes to isolate peripheral blood mononuclear cells (PBMCs) which were assessed for viability and cell count using a Vi-Cell automated cell counter (Beckman-Coulter). PBMCs were adjusted to 10 ×10^6^ cells/ml in cryopreservation media (90% FBS, 10% DMSO), frozen at −80°C using CoolCell LX (BioCision) for 12–24 hours and stored in liquid nitrogen vapor phase.

### RNA extraction, total RNA-seq, and analysis

RNA was extracted from 300K cells using the Zymo Direct-zol miniprep kit (Cat# R2051) and RNA quality assessed using the Agilent DNA tape screen assay. The RNA Integrity Number (RIN) scores for all samples were > 7.0. Total RNA libraries were generated using the NuGEN Ovation® SoLo RNA-Seq Library Preparation Kit (Cat# 0500–96). Libraries were sequenced using Illumina NovaSeq 6000 instrument with S4 flow cell and 150 base pair paired-end reads.FASTQ files were generated from the NovaSeq BCL outputs and quality was assessed with FASTQC^29^. Differentially expressed genes were identified between subjects with different disease severity using the limma package and voom to model variance^30^.

### Nuclei purification, ATAC-seq and analysis

Nuclei were extracted from frozen PBMCs. Briefly, 100K cells were spun down at 300 × g for 5 minutes at 4°C. The supernatant was removed, and cells were mixed with 100 uL of lysis buffer (10mM NaCl, 3mM MgCl2, 10mM Tris-HCl pH7.4, 0.1% Tween-20, 0.1% NonidetTM P40) and lysed on ice for 4 minutes. Wash buffer (1 mL; 10mM NaCl, 3mM MgCl2, 10mM Tris-HCl pH7.4, 0.1% Tween20) was added before centrifuging at 500 × g for 5 minutes at 4°C. ATAC-seq libraries were generated as described^14^. Briefly, transposition mix (25 μL 2× TD buffer, 2.5 μL transposase (Tn5, 100 nM final), 22.5 μL water) (Illumina Cat# 20031198) was added to the nuclear pellets, incubated at 37°C for 30 minutes, and samples purified using the Qiagen MinElute PCR Purification Kit (Qiagen Cat#28004). DNA fragments were PCR amplified for a total of 10–11 cycles and resulting libraries purified using the Qiagen MinElute PCR Purification Kit. The libraries were sequenced with an Illumina Novaseq 6000 S4 flow cell using 100 bp paired-end reads. FASTQ files were generated from the NovaSeq BCL outputs and used as input to the ENCODE ATAC-seq pipeline (https://github.com/ENCODE-DCC/atac-seq-pipeline) using the MACS2 peak-caller with all default parameters. Differential accessibility was calculated between groups of subjects with different disease severity using the csaw package^31^.

### Single-cell (sc)RNA-seq and analysis

Frozen PBMCs were thawed, and count and cell viability were measured by Countess II. The cell viability exceeded 80% for all samples except PBMC samples from CC subjects, which had viability between 70–80%. For single cell (sc) RNA-seq, 200K cells were aliquoted, spun down, resuspended in 30 uL PBS+0.04%BSA+0.2U / uL RNase inhibitor, and counted using Countess II. GEM generation, post GEMRT cleanup, cDNA amplification, and library construction were performed following 10X Genomics Single Cell 5’ v1 chemistry and quality was assessed using Agilent DNA tape screen assay. Libraries were then pooled and sequenced using Illumina NovaSeq 6000 platform with the goal of reaching saturation or 20,000 unique reads per cell on average. Sequencing data were used as input to the 10x Genomics Cell Ranger pipeline to demultiplex BCL files, generate FASTQs, and generate feature counts for each library. Dimensionality reduction and cell type annotation was accomplished using gene-barcode matrices generated using CellRanger count were analyzed using Seurat 3 with the default parameters unless otherwise specified^32^. Regulatory network inference was accomplished by converting the scRNA-seq Seurat object into a SingleCellExperiment and used as input to analysis with the SCENIC package^33^.

### scATAC-seq and analysis

PBMCs were thawed and nuclei were extracted as for ATAC-seq. The single-cell suspensions of scATAC-seq samples were converted to barcoded scATAC-seq libraries using the Chromium Single Cell 5′ Library, Gel Bead and Multiplex Kit, and Chip Kit (10x Genomics). The Chromium Single Cell 5′ v2 Reagent (10x Genomics, 120237) kit was used to prepare single-cell ATAC libraries according to the manufacturer’s instructions. Quality was assessed using Agilent DNA tape screen assay. Libraries were then pooled and sequenced using Illumina NovaSeq platform with the goal of reaching saturation or 25,000 unique reads per nuclei on average. Sequencing data were used as input to the 10x Genomics Cell Ranger ATAC pipeline to demultiplex BCL files, generate FASTQs, and generate feature counts for each library.scRNA-seq and scATAC-seq were integrated using fragment file outputs generated using CellRanger ATAC count were analyzed using ArchR following the standard workflow and with default parameters unless otherwise specified^34^. Feature and motif enrichment analysis (peak calling) was performed using MACS2 via the addReproduciblePeakSet() method in ArchR which uses pseudo-bulk replicates of cells grouped on a specific design variable. The correlations between chromVAR transcription factor deviation scores and scRNA-seq derived gene expression data were calculated using the ArchR method correlateMatrices() to identify activators and repressors. Topic-based clustering was performed for the CD14+ monocytes from the mild or moderate subject cohorts using the R package cisTopic^35^.

Detailed methods for qPCR and antibody tests, and epigenetic and genomic profiling, including RNA extraction, sequencing, differential expression analysis and differential chromatin accessibility analysis for both bulk and single cell analytic approaches can be found in Supplemental Methods.

## Supplementary Material

Supplement 1

## Figures and Tables

**Table 1:** Demographic characteristics and mean symptom severity scores of study participants.

A. Bulk assays for ATAC-seq and RNA-seq				
Characteristics	Healthy controls	Close contacts	Mild disease	Moderate disease
Number of subjects	7	7	7	7
				
Age Mean (range), yr	39.7 (25–61)	42.9 (17–61)	33.0 (27–60)	34.0 (20–52)
Sex (male/female)	3/4	4/3	4/3	3/4
Max severity score (±SE)	N/A	9.9 ±2.9	12.9 ±2.1	33.6 ±2.4
B. Single cell assays for ATAC-seq/RNA-seq				
Characteristics	Healthy controls	Close contacts	Mild disease	Moderate disease
Number of subjects	5	3	5	5
				
Age Mean (range), yr	45.2 (28–61)	53.2 (43–61)	35.6 (27–60)	37.7 (29–52)
Sex (male/female)	1/4	2/1	5/0	1/4
Max severity score (±SE)	N/A	3.3 ±2.8	12.2 ±2.8	36.0 ±2.3

## Data Availability

The sequencing datasets and related clinical metadata tables are available via the NIH/NCBI Gene Expression Omnibus (GEO) repository.

## References

[1] Blanco-Melo D. Imbalanced Host Response to SARS-CoV-2 Drives Development of COVID-19. Cell 181, 1036–1045 e1039, doi:10.1016/j.cell.2020.04.026 (2020). 32416070 PMC7227586

[2] Helling B. A. Altered transcriptional and chromatin responses to rhinovirus in bronchial epithelial cells from adults with asthma. Commun Biol 3, 678, doi:10.1038/s42003-020-01411-4 (2020). 33188283 PMC7666152

[3] Park A. & Iwasaki A. Type I and Type III Interferons - Induction, Signaling, Evasion, and Application to Combat COVID-19. Cell Host Microbe 27, 870–878, doi:10.1016/j.chom.2020.05.008 (2020). 32464097 PMC7255347

[4] Del Valle D. M. An inflammatory cytokine signature predicts COVID-19 severity and survival. Nat Med 26, 1636–1643, doi:10.1038/s41591-020-1051-9 (2020). 32839624 PMC7869028

[5] Schulte-Schrepping J. Severe COVID-19 Is Marked by a Dysregulated Myeloid Cell Compartment. Cell, doi:10.1016/j.cell.2020.08.001 (2020). PMC7405822 32810438

[6] Zhou Z. Heightened Innate Immune Responses in the Respiratory Tract of COVID-19 Patients. Cell Host & Microbe 27, 883–890.e882, doi:10.1016/j.chom.2020.04.017 (2020). 32407669 PMC7196896

[7] Ma S. Chromatin Potential Identified by Shared Single-Cell Profiling of RNA and Chromatin. Cell 183, 1103–1116 e1120, doi:10.1016/j.cell.2020.09.056 (2020). 33098772 PMC7669735

[8] Bournazos S., Gupta A. & Ravetch J. V. The role of IgG Fc receptors in antibody-dependent enhancement. Nat Rev Immunol 20, 633–643, doi:10.1038/s41577-020-00410-0 (2020). 32782358 PMC7418887

[9] Ibarrondo F. J. Rapid Decay of Anti-SARS-CoV-2 Antibodies in Persons with Mild Covid-19. N Engl J Med 383, 1085–1087, doi:10.1056/NEJMc2025179 (2020). 32706954 PMC7397184

[10] Long Q. X. Antibody responses to SARS-CoV-2 in patients with COVID-19. Nat Med 26, 845–848, doi:10.1038/s41591-020-0897-1 (2020). 32350462

[11] Zhao J. Antibody Responses to SARS-CoV-2 in Patients With Novel Coronavirus Disease 2019. Clin Infect Dis 71, 2027–2034, doi:10.1093/cid/ciaa344 (2020). 32221519 PMC7184337

[12] Amati A. L. Chemokines (CCL3, CCL4, and CCL5) Inhibit ATP-Induced Release of IL-1beta by Monocytic Cells. Mediators Inflamm 2017, 1434872, doi:10.1155/2017/1434872 (2017). 28757683 PMC5516742

[13] Goodman W. A. KLF6 contributes to myeloid cell plasticity in the pathogenesis of intestinal inflammation. Mucosal Immunol 9, 1250–1262, doi:10.1038/mi.2016.1 (2016). 26838049 PMC4972715

[14] Buenrostro J. D., Wu B., Chang H. Y. & Greenleaf W. J. ATAC-seq: A Method for Assaying Chromatin Accessibility Genome-Wide. Curr Protoc Mol Biol 109, 21 29 21–21 29 29, doi:10.1002/0471142727.mb2129s109 (2015). PMC4374986 25559105

[15] Alrdahe S. Dysregulation of macrophage development and phenotype in diabetic human macrophages can be rescued by Hoxa3 protein transduction. PLoS One 14, e0223980, doi:10.1371/journal.pone.0223980 (2019). 31626638 PMC6799902

[16] Beutler B. A. TLRs and innate immunity. Blood 113, 1399–1407, doi:10.1182/blood-2008-07-019307 (2009). 18757776 PMC2644070

[17] Brown G. D. Dectin-1: a signalling non-TLR pattern-recognition receptor. Nat Rev Immunol 6, 33–43, doi:10.1038/nri1745 (2006). 16341139

[18] Niu X. Longitudinal Analysis of T and B Cell Receptor Repertoire Transcripts Reveal Dynamic Immune Response in COVID-19 Patients. Front Immunol 11, 582010, doi:10.3389/fimmu.2020.582010 (2020). 33117392 PMC7561365

[19] julian.knight@well.ox.ac.uk, C. O.-M.-o. B. A. C. E. a. & Consortium, C. O.-M.-o. B. A. A blood atlas of COVID-19 defines hallmarks of disease severity and specificity. Cell 185, 916–938 e958, doi:10.1016/j.cell.2022.01.012 (2022). 35216673 PMC8776501

[20] Li S. Epigenetic Landscapes of Single-Cell Chromatin Accessibility and Transcriptomic Immune Profiles of T Cells in COVID-19 Patients. Front Immunol 12, 625881, doi:10.3389/fimmu.2021.625881 (2021). 33717140 PMC7943924

[21] Wilk A. J. Multi-omic profiling reveals widespread dysregulation of innate immunity and hematopoiesis in COVID-19. J Exp Med 218, doi:10.1084/jem.20210582 (2021). PMC8210586 34128959

[22] You M. Single-cell epigenomic landscape of peripheral immune cells reveals establishment of trained immunity in individuals convalescing from COVID-19. Nat Cell Biol 23, 620–630, doi:10.1038/s41556-021-00690-1 (2021). 34108657 PMC9105401

[23] Kapoor N. Transcription factors STAT6 and KLF4 implement macrophage polarization via the dual catalytic powers of MCPIP. J Immunol 194, 6011–6023, doi:10.4049/jimmunol.1402797 (2015). 25934862 PMC4458412

[24] Sweet D. R., Fan L., Hsieh P. N. & Jain M. K. Kruppel-Like Factors in Vascular Inflammation: Mechanistic Insights and Therapeutic Potential. Front Cardiovasc Med 5, 6, doi:10.3389/fcvm.2018.00006 (2018). 29459900 PMC5807683

[25] Konigsberg I. R. Host methylation predicts SARS-CoV-2 infection and clinical outcome. Commun Med (London) 1, 42, doi:10.1038/s43856-021-00042-y (2021). 35072167 PMC8767772

[26] Corley M. J. Genome-wide DNA methylation profiling of peripheral blood reveals an epigenetic signature associated with severe COVID-19. J Leukoc Biol 110, 21–26, doi:10.1002/JLB.5HI0720-466R (2021). 33464637 PMC8013321

[27] Altschul D. J. A novel severity score to predict inpatient mortality in COVID-19 patients. Sci Rep 10, 16726, doi:10.1038/s41598-020-73962-9 (2020). 33028914 PMC7542454

[28] Cote A., Ternacle J. & Pibarot P. Early prediction of the risk of severe coronavirus disease 2019: A key step in therapeutic decision making. EBioMedicine 59, 102948, doi:10.1016/j.ebiom.2020.102948 (2020). 32810827 PMC7428762

[29] Andrews S. FastQC: A Quality Control Tool for High Throughput Sequence Data [Online], <https://www.bioinformatics.babraham.ac.uk/projects/fastqc/> (2015).

[30] Law C. W., Chen Y., Shi W. & Smyth G. K. voom: Precision weights unlock linear model analysis tools for RNA-seq read counts. Genome Biol 15, R29, doi:10.1186/gb-2014-15-2-r29 (2014). 24485249 PMC4053721

[31] Lun A. T. & Smyth G. K. csaw: a Bioconductor package for differential binding analysis of ChIP-seq data using sliding windows. Nucleic Acids Res 44, e45, doi:10.1093/nar/gkv1191 (2016). 26578583 PMC4797262

[32] Stuart T. Comprehensive Integration of Single-Cell Data. Cell 177, 1888–1902.e1821, doi:10.1016/j.cell.2019.05.031 (2019). 31178118 PMC6687398

[33] Aibar S. SCENIC: single-cell regulatory network inference and clustering. Nature Methods 14, 1083–1086, doi:10.1038/nmeth.4463 (2017). 28991892 PMC5937676

[34] Granja J. M. ArchR is a scalable software package for integrative single-cell chromatin accessibility analysis. Nat Genet 53, 403–411, doi:10.1038/s41588-021-00790-6 (2021). 33633365 PMC8012210

[35] Bravo Gonzalez-Blas C. cisTopic: cis-regulatory topic modeling on single-cell ATAC-seq data. Nat Methods 16, 397–400, doi:10.1038/s41592-019-0367-1 (2019). 30962623 PMC6517279

