# Supplementary material for: Differential chromatin accessibility in peripheral blood mononuclear cells underlies COVID-19 disease severity prior to seroconversion
Source: Res Sq. 2022 Apr 7:rs.3.rs-1479864. Preprint. [Version 1] doi: 10.21203/rs.3.rs-1479864/v1 (PMC8996625; doi:10.21203/rs.3.rs-1479864/v1)
Supplement: Supplement 1 [file f1efa2eae52e54f0841d0792.docx]

**SUPPLEMENTAL INFORMATION**


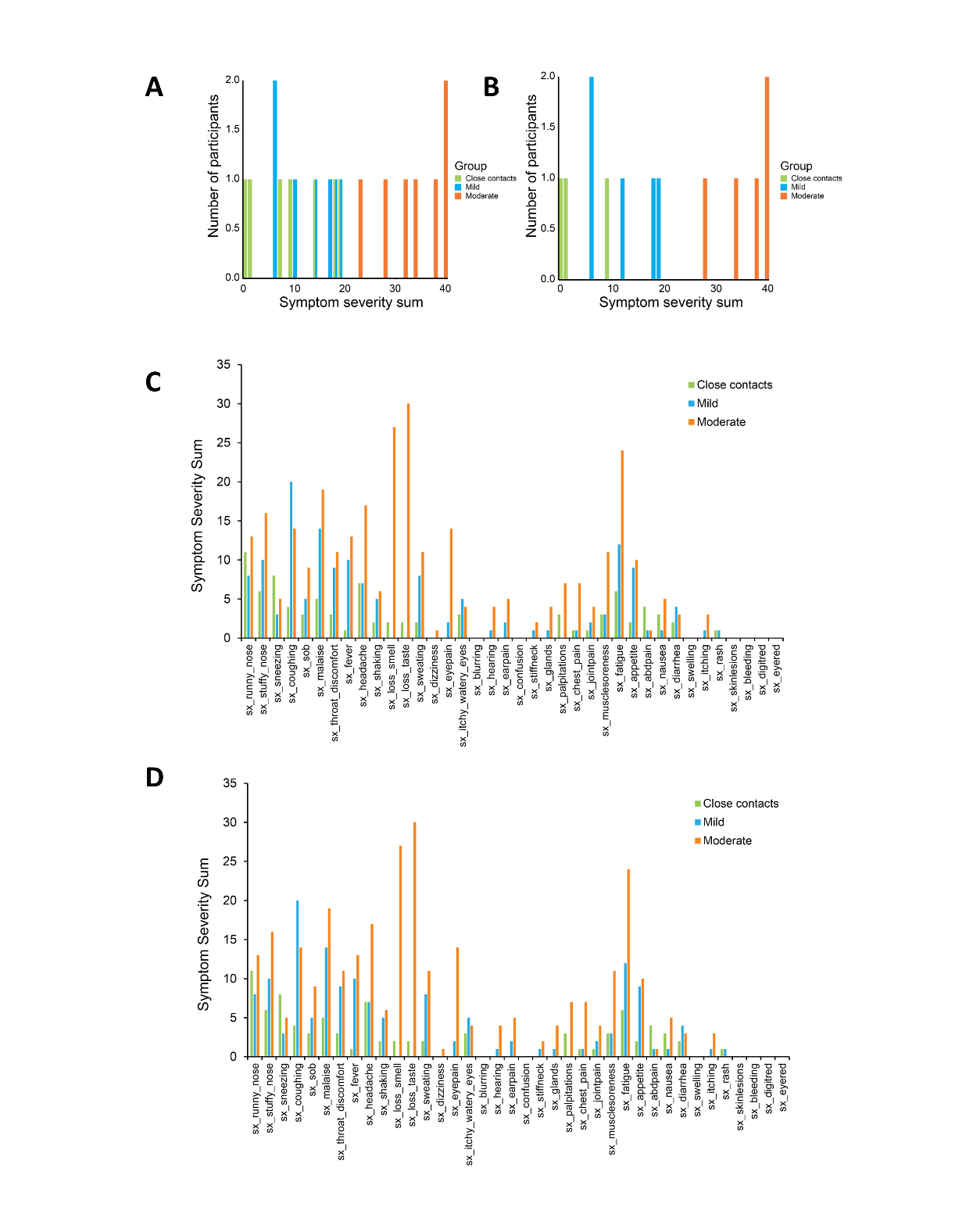


**Supplemental Figure S1. (A)** Symptom severity sums for the subjects profiled with bulk assays and **(B)** single-cell assays. The sum of severity scores for all reported symptoms are shown for close contacts (green), COVID-19 subjects with mild symptoms (blue), and with moderate symptoms (orange). **(C)** Severity scores for each reported symptom for the subjects profiled with bulk assays and **(D)** single-cell assays. The sum of severity scores for each reported symptom are shown for close contacts (green), COVID-19 subjects with mild symptoms (blue), and with moderate symptoms (orange).

**Supplemental Data Table 1**. Subject serology and PCR results on days when PBMCs were collected for bulk assays.

| **Group** | **Alias ID** | **Race** | **Ethnicity** | **Day of PBMC collection** | | | **PCR** | | | **Serology Test (IgG)** | | |
| --- | --- | --- | --- | --- | --- | --- | --- | --- | --- | --- | --- | --- |
| **Healthy** | 22CBD6 | White | NR | N/A | | | N/A | | | N/A | | |
|  | 77DA77 | White | NR | N/A | | | N/A | | | N/A | | |
|  | 4F2EAD | White | NR | N/A | | | N/A | | | N/A | | |
|  | 1FD3AC | White | NR | N/A | | | N/A | | | N/A | | |
|  | F8FF1A | White | NR | N/A | | | N/A | | | N/A | | |
|  | 56E219 | Black | NR | N/A | | | N/A | | | N/A | | |
|  | 3F3832 | White | NR | N/A | | | N/A | | | N/A | | |
| **Close contacts** | 7768E4 | NR | Non-Hispanic | 2 | 14 | 28 | N | N | N | N | N | N |
|  | 61BBAD | White | Non-Hispanic | 0 | 7 | 14 | N | N | N | N | N | N |
|  | 80A16A | Asian | Non-Hispanic | 0 | 14 |  | N | N |  | N | N |  |
|  | CE0CE8 | White | Non-Hispanic | 0 | 7 | 14 | N | N | N | N | N | N |
|  | 5ABDCB | White | Non-Hispanic | 0 | 14 | 28 | N | N | N | N | N | N |
|  | B96D3B | White | Hispanic | 0 | 14 | 28 | N | N | N | N | N | N |
|  | 6F894A | White | Non-Hispanic | 0 | 7 | 14 | N | N | N | N | N | N |
| **Mild** | 0B943B | White | Non-Hispanic | 0 | 3 | 14 | P | P | P | N | N | P |
|  | 2AD75E | Asian | Non-Hispanic | 0 | 7 | 14 | N^‡^ | N | N | N | N | BP |
|  | 450905 | White | Non-Hispanic | 0 | 7 | 14 | P | P | P | N | P | P |
|  | BAAF62 | White | Non-Hispanic | 0 | 7 | 14 | P | P | P | N | P | P |
|  | 1A9B20 | White | Non-Hispanic | 0 | 7 | 14 | P | P | P | N | P | P |
|  | 180E1A | White | Non-Hispanic | 0 | 7 | 14 | P | P | P | N | P | P |
|  | 82CCF5 | White | Non-Hispanic | 0 | 7 | 14 | P | P | P | N | P | P |
|  | 75A2B6 | Asian | Non-Hispanic | 0 | 7 | 14 | P | P | P | N | P | P |
|  | DF309F | White | Hispanic | 0 | 7 | 14 | N^‡^ | N | N | N | P | P |
| **Moderate** | 180E1A | White | Non-Hispanic | 0 | 7 | 14 | P | N | N | N | P | P |
|  | 82CCF5 | White | Non-Hispanic | 0 | 7 | 14 | P | P | P | N | P | P |
|  | B85D75 | White | Non-Hispanic | 0 | 7 |  | P | P |  | N | P |  |
|  | 40067F | White | Non-Hispanic | 0 | 14 | 21 | P | P | P | N | N | P |
|  | 0BF51C | White | Non-Hispanic | 0 | 7 | 14 | P | P | P | N | P | NR |
|  | 0E1F8E | White | Non-Hispanic | 0 | 7 | 14 | P | P | P | N | P | P |
|  | 3F05F3 | White | Non-Hispanic | 0 | 7 | 14 | P | P | N | N | P | P |

NR, not reported

N, negative; P, positive; BP, borderline positive

^‡^ Positive clinical PCR prior to enrollment, but negative research qPCR

**Supplemental Data Table 2**. Subject serology and PCR results on days when PBMCs collected for single-cell assays.

| **Group** | **Alias ID** | **Race** | **Ethnicity** | **Day of PBMC collection** | | | **PCR** | | | **Serology Test (IgG)** | | |
| --- | --- | --- | --- | --- | --- | --- | --- | --- | --- | --- | --- | --- |
| **Healthy** | 22CBD6 | White | NR | N/A | | | N/A | | | N/A | | |
|  | 77DA77 | White | NR | N/A | | | N/A | | | N/A | | |
|  | 4F2EAD | White | NR | N/A | | | N/A | | | N/A | | |
|  | F8FF1A | White | NR | N/A | | | N/A | | | N/A | | |
|  | 56E219 | Black | NR | N/A | | | N/A | | | N/A | | |
| **Close contacts** | 7768E4 | NR | Non-Hispanic | 2 | 14 | 28 | N | N | N | N | N | N |
|  | 61BBAD | White | Non-Hispanic | 0 | 7 | 14 | N | N | N | N | N | N |
|  | 80A16A | Asian | Non-Hispanic | 0 | 14 |  | N | N |  | N | N |  |
| **Mild** | 0B943B | White | Non-Hispanic | 0 | 3 | 14 | P | P | P | N | N | P |
|  | 7085CA | White | Non-Hispanic | 0 | 7 | 14 | N^‡^ | N | N | N | N | P |
|  | 2AD75E | Asian | Non-Hispanic | 0 | 7 | 14 | N^‡^ | N | N | N | N | BP |
|  | 450905 | White | Non-Hispanic | 0 | 7 | 14 | P | P | P | N | P | P |
|  | BAAF62 | White | Non-Hispanic | 0 | 7 | 14 | P | P | P | N | P | P |
| **Moderate** | 180E1A | White | Non-Hispanic | 0 | 7 | 14 | P | N | N | N | P | P |
|  | 82CCF5 | White | Non-Hispanic | 0 | 7 | 14 | P | P | P | N | P | P |
|  | B85D75 | White | Non-Hispanic | 0 | 7 |  | P | P |  | N | P |  |
|  | 40067F | White | Non-Hispanic | 0 | 14 | 21 | P | P | P | N | N | P |
|  | 0BF51C | White | Non-Hispanic | 0 | 7 | 14 | P | P | P | N | P | NR |

NR, not reported

N, negative; P, positive; BP, borderline positive

^‡^ Positive clinical PCR prior to enrollment, but negative research qPCR

**Supplemental Data Table 3** Gene symbols, gene names, fold change and p values adjusted for multiple hypothesis testing p <= 0.05 for RNA-seq of the four comparisons shown in Figure 1.

| **Gene Symbol** | **Fold Change (log2 )** | **padj** | **Gene Name** | **Gene Alias** |
| --- | --- | --- | --- | --- |
| **IgG Negative Healthy vs Mild** | | | | |
| CXCL8 | 4.647 | 0.000 | C-X-C motif chemokine ligand 8 | GCP-1, GCP1, IL8, LECT, LUCT |
| RGS2 | 1.647 | 0.000 | regulator of G protein signaling 2 | G0S8 |
| CXCL2 | 4.948 | 0.001 | C-X-C motif chemokine ligand 2 | CINC-2a, GRO2, GROb, MGSA-b, MIP-2a |
| G0S2 | 3.752 | 0.002 | G0/G1 switch 2 |  |
| CXCR1 | -1.917 | 0.004 | C-X-C motif chemokine receptor 1 | C-C, C-C-CKR-1, CD128, CD181, CDw128a |
| HCAR3 | 1.921 | 0.004 | hydroxycarboxylic acid receptor 3 | GPR109B, HCA3, HM74, PUMAG, Puma-g |
| NR4A2 | 2.599 | 0.004 | nuclear receptor subfamily 4 group A member 2 | HZF-3, NOT, NURR1, RNR1, TINUR |
| PDE4B | 1.591 | 0.004 | phosphodiesterase 4B | DPDE4, PDEIVB |
| CXCR2 | -1.590 | 0.007 | C-X-C motif chemokine receptor 2 | CD182, CDw128b, CMKAR2, IL8R2, IL8RA |
| HERPUD1 | 0.897 | 0.007 | homocysteine inducible ER protein with ubiquitin like domain 1 | HERP, Mif1, SUP |
| NR4A3 | 3.552 | 0.007 | nuclear receptor subfamily 4 group A member 3 | CHN, CSMF, MINOR, NOR1 |
| TGIF1 | 0.912 | 0.009 | TGFB induced factor homeobox 1 | HPE4, TGIF |
| IL1B | 2.911 | 0.010 | interleukin 1 beta | IL-1, IL1-BETA, IL1F2, IL1beta |
| HCAR2 | 1.947 | 0.010 | hydroxycarboxylic acid receptor 2 | GPR109A, HCA2, HM74a, HM74b, NIACR1 |
| GPR183 | 1.216 | 0.011 | G protein-coupled receptor 183 | EBI2, hEBI2 |
| KLF6 | -0.770 | 0.011 | Kruppel like factor 6 | BCD1, CBA1, COPEB, CPBP, GBF |
| LIPN | 1.582 | 0.011 | lipase family member N | ARCI8, LI4, LIPL4, bA186O14.3 |
| PTGER4 | 0.913 | 0.011 | prostaglandin E receptor 4 | EP4, EP4R |
| HBB | 6.614 | 0.011 | hemoglobin subunit beta | CD113t-C, ECYT6, beta-globin |
| PLK2 | 2.255 | 0.011 | polo like kinase 2 | SNK, hPlk2, hSNK |
| SEC14L2 | 1.796 | 0.011 | SEC14 like lipid binding 2 | C22orf6, SPF, TAP, TAP1 |
| AREG | 2.302 | 0.017 | amphiregulin | AR, AREGB, CRDGF, SDGF |
| ASTL | 3.762 | 0.017 | astacin like metalloendopeptidase | OOMD11, SAS1B |
| CD83 | 1.708 | 0.017 | CD83 molecule | BL11, HB15 |
| CDKN1B | 0.699 | 0.017 | cyclin dependent kinase inhibitor 1B | CDKN4, KIP1, MEN1B, MEN4, P27KIP1 |
| SLC5A6 | 0.914 | 0.017 | solute carrier family 5 member 6 | NERIB, SMVT |
| FOS | 1.151 | 0.018 | Fos proto-oncogene, AP-1 transcription factor subunit | AP-1, C-FOS, p55 |
| SOD2 | 0.852 | 0.018 | superoxide dismutase 2 | GClnc1, IPO-B, IPOB, MNSOD, MVCD6 |
| JUNB | 1.626 | 0.019 | JunB proto-oncogene, AP-1 transcription factor subunit | AP-1 |
| CCL3 | 1.891 | 0.022 | C-C motif chemokine ligand 3 | G0S19-1, LD78ALPHA, MIP-1-alpha, MIP1A, SCYA3 |
| CREM | 1.511 | 0.023 | cAMP responsive element modulator | CREM-2, ICER, hCREM-2 |
| FBXO32 | 0.789 | 0.024 | F-box protein 32 | Fbx32, MAFbx |
| IRS2 | 1.278 | 0.024 | insulin receptor substrate 2 | IRS-2 |
| TP53INP2 | 1.692 | 0.032 | tumor protein p53 inducible nuclear protein 2 | C20orf110, DOR, PIG-U, PIGU, PINH |
| CD55 | 1.064 | 0.033 | CD55 molecule (Cromer blood group) | CHAPLE, CR, CROM, DAF, TC |
| NUDT15 | 0.936 | 0.035 | nudix hydrolase 15 | MTH2, NUDT15D |
| CXCL1 | 2.158 | 0.035 | C-X-C motif chemokine ligand 1 | FSP, GRO1, GROa, MGSA, MGSA-a |
| CXCR4 | 1.742 | 0.037 | C-X-C motif chemokine receptor 4 | CD184, D2S201E, FB22, HM89, HSY3RR |
| DNAJB9 | 1.060 | 0.037 | DnaJ heat shock protein family (Hsp40) member B9 | ERdj4, MDG-1, MDG1, MST049, MSTP049 |
| OLR1 | 4.208 | 0.043 | oxidized low density lipoprotein receptor 1 | CLEC8A, LOX1, LOXIN, SCARE1, SLOX1 |
| CXCL3 | 3.227 | 0.050 | C-X-C motif chemokine ligand 3 | CINC-2b, GRO3, GROg, MIP-2b, MIP2B |
| LGALS9C | -1.784 | 0.050 | galectin 9C | Gal-9B, LGALS9B |
| NAMPT | 2.196 | 0.050 | nicotinamide phosphoribosyltransferase | 1110035O14Rik, PBEF, PBEF1, VF, VISFATIN |
| NR4A1 | 2.518 | 0.050 | nuclear receptor subfamily 4 group A member 1 | GFRP1, HMR, N10, NAK-1, NGFIB |
| RIPK2 | 1.026 | 0.050 | receptor interacting serine/threonine kinase 2 | CARD3, CARDIAK, CCK, GIG30, RICK |
|  |  |  |  |  |
| **IgG Negative Healthy vs Moderate** | | | | |
| CXCL8 | 4.864 | 0.000 | C-X-C motif chemokine ligand 8 | GCP-1, GCP1, IL8, LECT, LUCT |
| RGS2 | 2.010 | 0.000 | regulator of G protein signaling 2 | G0S8 |
| HCAR2 | 3.169 | 0.002 | hydroxycarboxylic acid receptor 2 | GPR109A, HCA2, HM74a, HM74b, NIACR1 |
| CXCL2 | 4.822 | 0.007 | C-X-C motif chemokine ligand 2 | CINC-2a, GRO2, GROb, MGSA-b, MIP-2a |
| GPR183 | 1.453 | 0.007 | G protein-coupled receptor 183 | EBI2, hEBI2 |
| HCAR3 | 2.236 | 0.007 | hydroxycarboxylic acid receptor 3 | GPR109B, HCA3, HM74, PUMAG, Puma-g |
| HJURP | -2.710 | 0.009 | Holliday junction recognition protein | FAKTS, URLC9, hFLEG1 |
| PDE4B | 1.525 | 0.012 | phosphodiesterase 4B | DPDE4, PDEIVB |
| PTGER4 | 0.996 | 0.012 | prostaglandin E receptor 4 | EP4, EP4R |
| UHRF1 | -1.796 | 0.012 | ubiquitin like with PHD and ring finger domains 1 | ICBP90, Np95, RNF106, TDRD22, hNP95 |
| IL1B | 3.373 | 0.013 | interleukin 1 beta | IL-1, IL1-BETA, IL1F2, IL1beta |
| FBXO32 | 0.962 | 0.014 | F-box protein 32 | Fbx32, MAFbx |
| CDKN1B | 0.778 | 0.015 | cyclin dependent kinase inhibitor 1B | CDKN4, KIP1, MEN1B, MEN4, P27KIP1 |
| HBB | 6.876 | 0.015 | hemoglobin subunit beta | CD113t-C, ECYT6, beta-globin |
| FOS | 1.240 | 0.016 | Fos proto-oncogene, AP-1 transcription factor subunit | AP-1, C-FOS, p55 |
| CXCR2 | -1.580 | 0.018 | C-X-C motif chemokine receptor 2 | CD182, CDw128b, CMKAR2, IL8R2, IL8RA |
| B4GALT3 | -0.816 | 0.019 | beta-1,4-galactosyltransferase 3 | beta4Gal-T3 |
| NR4A2 | 2.302 | 0.019 | nuclear receptor subfamily 4 group A member 2 | HZF-3, NOT, NURR1, RNR1, TINUR |
| CCL3 | 2.185 | 0.028 | C-C motif chemokine ligand 3 | G0S19-1, LD78ALPHA, MIP-1-alpha, MIP1A, SCYA3 |
| KLF6 | -0.721 | 0.028 | Kruppel like factor 6 | BCD1, CBA1, COPEB, CPBP, GBF |
| PLK2 | 2.415 | 0.028 | polo like kinase 2 | SNK, hPlk2, hSNK |
| CREBRF | 0.747 | 0.028 | CREB3 regulatory factor | C5orf41, LRF |
| AREG | 2.637 | 0.028 | amphiregulin | AR, AREGB, CRDGF, SDGF |
| CCNB2 | -2.059 | 0.028 | cyclin B2 | HsT17299 |
| FOXM1 | -1.766 | 0.028 | forkhead box M1 | FKHL16, FOXM1A, FOXM1B, FOXM1C, HFH-11 |
| PIK3R1 | 1.211 | 0.028 | phosphoinositide-3-kinase regulatory subunit 1 | AGM7, GRB1, IMD36, p85, p85-ALPHA |
| RGS3 | -1.185 | 0.028 | regulator of G protein signaling 3 | C2PA, RGP3 |
| CD55 | 1.144 | 0.029 | CD55 molecule (Cromer blood group) | CHAPLE, CR, CROM, DAF, TC |
| SLC19A2 | 1.464 | 0.029 | solute carrier family 19 member 2 | TC1, THMD1, THT1, THTR1, TRMA |
| CDCA3 | -1.886 | 0.031 | cell division cycle associated 3 | GRCC8, TOME-1, TOME1 |
| CXCR1 | -1.565 | 0.031 | C-X-C motif chemokine receptor 1 | C-C, C-C-CKR-1, CD128, CD181, CDw128a |
| MKI67 | -1.859 | 0.031 | marker of proliferation Ki-67 | KIA, MIB-, MIB-1, PPP1R105 |
| MLLT3 | 1.060 | 0.031 | MLLT3 super elongation complex subunit | AF9, YEATS3 |
| SLC4A1 | 5.526 | 0.031 | solute carrier family 4 member 1 (Diego blood group) | AE1, BND3, CD233, CHC, DI |
| TUBB | -0.571 | 0.031 | tubulin beta class I | CDCBM6, CSCSC1, M40, OK/SW-cl.56, TUBB1 |
| KIF2C | -1.660 | 0.031 | kinesin family member 2C | CT139, KNSL6, MCAK |
| TYMS | -2.014 | 0.031 | thymidylate synthetase | HST422, TMS, TS |
| LIPN | 1.495 | 0.033 | lipase family member N | ARCI8, LI4, LIPL4, bA186O14.3 |
| PDIK1L | 0.730 | 0.033 | PDLIM1 interacting kinase 1 like | CLIK1L, STK35L2 |
| TGIF1 | 0.785 | 0.034 | TGFB induced factor homeobox 1 | HPE4, TGIF |
| VNN3 | 1.840 | 0.037 | vanin 3, pseudogene | HSA238982, VNN3 |
| DNAJB9 | 1.128 | 0.037 | DnaJ heat shock protein family (Hsp40) member B9 | ERdj4, MDG-1, MDG1, MST049, MSTP049 |
| ESPL1 | -1.693 | 0.037 | extra spindle pole bodies like 1, separase | ESP1, SEPA |
| ADAM8 | -1.089 | 0.038 | ADAM metallopeptidase domain 8 | CD156, CD156a, MS2 |
| CITED2 | 0.797 | 0.038 | Cbp/p300 interacting transactivator with Glu/Asp rich carboxy-terminal domain 2 | ASD8, MRG-1, MRG1, P35SRJ, VSD2 |
| CXCR4 | 1.740 | 0.038 | C-X-C motif chemokine receptor 4 | CD184, D2S201E, FB22, HM89, HSY3RR |
| G0S2 | 2.482 | 0.038 | G0/G1 switch 2 |  |
| HERPUD1 | 0.730 | 0.038 | homocysteine inducible ER protein with ubiquitin like domain 1 | HERP, Mif1, SUP |
| LYPD3 | 1.784 | 0.038 | LY6/PLAUR domain containing 3 | C4.4A |
| MCM10 | -1.738 | 0.038 | minichromosome maintenance 10 replication initiation factor | CNA43, DNA43, IMD80, PRO2249 |
| NR4A3 | 2.962 | 0.038 | nuclear receptor subfamily 4 group A member 3 | CHN, CSMF, MINOR, NOR1 |
| NSA2 | 0.674 | 0.038 | NSA2 ribosome biogenesis factor | CDK105, HCL-G1, HCLG1, HUSSY-29, HUSSY29 |
| SOD2 | 0.789 | 0.038 | superoxide dismutase 2 | GClnc1, IPO-B, IPOB, MNSOD, MVCD6 |
| SSTR3 | -1.493 | 0.038 | somatostatin receptor 3 | SS-3-R, SS3-R, SS3R, SSR-28 |
| TOPORS | 0.764 | 0.038 | TOP1 binding arginine/serine rich protein, E3 ubiquitin ligase | LUN, P53BP3, RP31, TP53BPL |
| TPT1 | 0.682 | 0.038 | tumor protein, translationally-controlled 1 | HRF, TCTP, p02, p23 |
| ZBTB6 | 1.024 | 0.038 | zinc finger and BTB domain containing 6 | ZID, ZNF482 |
| PNPO | -0.663 | 0.038 | pyridoxamine 5'-phosphate oxidase | HEL-S-302, PDXPO |
| LGALS9C | -1.873 | 0.040 | galectin 9C | Gal-9B, LGALS9B |
| MPHOSPH10 | 0.662 | 0.040 | M-phase phosphoprotein 10 | CT90, MPP10, MPP10P, PPP1R106 |
| CHAF1A | -0.651 | 0.041 | chromatin assembly factor 1 subunit A | CAF-1, CAF1, CAF1B, CAF1P150, P150 |
| PPP2R3A | 1.076 | 0.041 | protein phosphatase 2 regulatory subunit B''alpha | PPP2R3, PR130, PR72 |
| RNF144B | 1.241 | 0.041 | ring finger protein 144B | IBRDC2, PIR2, bA528A10.3, p53RFP |
| ANKRD55 | 1.225 | 0.042 | ankyrin repeat domain 55 |  |
| MYBL2 | -1.668 | 0.042 | MYB proto-oncogene like 2 | B-MYB, BMYB |
| ZFP36L2 | 0.983 | 0.042 | ZFP36 ring finger protein like 2 | BRF2, ERF-2, ERF2, RNF162C, TIS11D |
| ZNF277 | 0.883 | 0.043 | zinc finger protein 277 | NRIF4, ZNF277P |
| APOBR | -1.094 | 0.043 | apolipoprotein B receptor | APOB100R, APOB48R |
| ASB1 | -0.604 | 0.043 | ankyrin repeat and SOCS box containing 1 | ASB-1 |
| CHST12 | -0.959 | 0.043 | carbohydrate sulfotransferase 12 | C4S-2, C4ST-2, C4ST2 |
| SERPINB2 | 3.087 | 0.043 | serpin family B member 2 | HsT1201, PAI, PAI-2, PAI2, PLANH2 |
|  |  |  |  |  |
| **IgG Positive Healthy vs Mild** | | | | |
| ABCA1 | 1.080 | 0.027 | ATP binding cassette subfamily A member 1 | ABC-1, ABC1, CERP, HDLCQTL13, HDLDT1 |
| ACSL1 | 1.313 | 0.005 | acyl-CoA synthetase long chain family member 1 | ACS1, FACL1, FACL2, LACS, LACS1 |
| ADIPOR1 | 0.372 | 0.044 | adiponectin receptor 1 | ACDCR1, CGI-45, CGI45, PAQR1, TESBP1A |
| ADNP2 | 0.609 | 0.022 | ADNP homeobox 2 | ZNF508 |
| AKAP8 | 0.458 | 0.039 | A-kinase anchoring protein 8 | AKAP 95, AKAP-8, AKAP-95, AKAP95 |
| ALAS2 | 5.523 | 0.019 | 5'-aminolevulinate synthase 2 | ALAS-E, ALASE, ANH1, ASB, SIDBA1 |
| ANK3 | 0.763 | 0.042 | ankyrin 3 | ANKYRIN-G, MRT37 |
| AREG | 2.310 | 0.001 | amphiregulin | AR, AREGB, CRDGF, SDGF |
| ARHGEF2 | -0.460 | 0.050 | Rho/Rac guanine nucleotide exchange factor 2 | GEF, GEF-H1, GEFH1, LFP40, Lfc |
| ARL4A | 0.647 | 0.036 | ADP ribosylation factor like GTPase 4A | ARL4 |
| ARL5B | 0.900 | 0.032 | ADP ribosylation factor like GTPase 5B | ARL8 |
| ASTL | 2.763 | 0.013 | astacin like metalloendopeptidase | OOMD11, SAS1B |
| ATF3 | 2.125 | 0.002 | activating transcription factor 3 |  |
| AVPI1 | 1.236 | 0.044 | arginine vasopressin induced 1 | PP5395, VIP32, VIT32 |
| BCL2A1 | 1.391 | 0.003 | BCL2 related protein A1 | ACC-1, ACC-2, ACC1, ACC2, BCL2L5 |
| BCL2L11 | 0.631 | 0.019 | BCL2 like 11 | BAM, BIM, BOD |
| BEST1 | 1.120 | 0.017 | bestrophin 1 | ARB, BEST, BMD, Best1V1Delta2, RP50 |
| BLCAP | -0.407 | 0.038 | BLCAP apoptosis inducing factor | BC10 |
| BTBD1 | 0.455 | 0.040 | BTB domain containing 1 | C15orf1, NS5ATP8 |
| BTN2A2 | -0.756 | 0.006 | butyrophilin subfamily 2 member A2 | BT2.2, BTF2, BTN2.2 |
| C17orf107 | 1.442 | 0.008 | chromosome 17 open reading frame 107 |  |
| C1orf216 | -0.659 | 0.046 | chromosome 1 open reading frame 216 |  |
| C5orf63 | -0.790 | 0.031 | chromosome 5 open reading frame 63 | YDR286C |
| C6orf47 | -0.659 | 0.038 | chromosome 6 open reading frame 47 | D6S53E, G4, NG34 |
| CATSPERD | 2.695 | 0.032 | cation channel sperm associated auxiliary subunit delta | TMEM146 |
| CCL3 | 2.664 | 0.000 | C-C motif chemokine ligand 3 | G0S19-1, LD78ALPHA, MIP-1-alpha, MIP1A, SCYA3 |
| CCNH | 0.909 | 0.003 | cyclin H | CAK, CycH, p34, p37 |
| CD55 | 1.055 | 0.002 | CD55 molecule (Cromer blood group) | CHAPLE, CR, CROM, DAF, TC |
| CD83 | 1.836 | 0.000 | CD83 molecule | BL11, HB15 |
| CDK6 | 0.569 | 0.038 | cyclin dependent kinase 6 | MCPH12, PLSTIRE |
| CDKN1A | 1.319 | 0.007 | cyclin dependent kinase inhibitor 1A | CAP20, CDKN1, CIP1, MDA-6, P21 |
| CDKN1B | 0.602 | 0.009 | cyclin dependent kinase inhibitor 1B | CDKN4, KIP1, MEN1B, MEN4, P27KIP1 |
| CEBPZ | 0.603 | 0.033 | CCAAT enhancer binding protein zeta | CBF, CBF2, HSP-CBF, NOC1 |
| CHD1 | 0.856 | 0.047 | chromodomain helicase DNA binding protein 1 | CHD-1, PILBOS |
| CHMP1B | 0.582 | 0.009 | charged multivesicular body protein 1B | C10orf2, C18-ORF2, C18orf2, CHMP1.5, Vps46-2 |
| CLEC7A | 0.717 | 0.032 | C-type lectin domain containing 7A | BGR, CANDF4, CD369, CLECSF12, DECTIN1 |
| CLIC3 | -1.377 | 0.011 | chloride intracellular channel 3 |  |
| CLK4 | 0.534 | 0.034 | CDC like kinase 4 |  |
| COPS7B | -0.442 | 0.031 | COP9 signalosome subunit 7B | CSN7B, SGN7b |
| COQ10B | 0.605 | 0.046 | coenzyme Q10B |  |
| CREBRF | 0.640 | 0.022 | CREB3 regulatory factor | C5orf41, LRF |
| CRELD1 | -0.626 | 0.044 | cysteine rich with EGF like domains 1 | AVSD2, CIRRIN |
| CREM | 1.616 | 0.005 | cAMP responsive element modulator | CREM-2, ICER, hCREM-2 |
| CTNNB1 | 0.360 | 0.040 | catenin beta 1 | CTNNB, EVR7, MRD19, NEDSDV, armadillo |
| CXCL1 | 2.574 | 0.005 | C-X-C motif chemokine ligand 1 | FSP, GRO1, GROa, MGSA, MGSA-a |
| CXCL2 | 4.632 | 0.000 | C-X-C motif chemokine ligand 2 | CINC-2a, GRO2, GROb, MGSA-b, MIP-2a |
| CXCL3 | 3.617 | 0.002 | C-X-C motif chemokine ligand 3 | CINC-2b, GRO3, GROg, MIP-2b, MIP2B |
| CXCL8 | 5.846 | 0.000 | C-X-C motif chemokine ligand 8 | GCP-1, GCP1, IL8, LECT, LUCT |
| CXCR1 | -1.433 | 0.049 | C-X-C motif chemokine receptor 1 | C-C, C-C-CKR-1, CD128, CD181, CDw128a |
| CXCR2 | -1.384 | 0.008 | C-X-C motif chemokine receptor 2 | CD182, CDw128b, CMKAR2, IL8R2, IL8RA |
| CXCR4 | 1.459 | 0.009 | C-X-C motif chemokine receptor 4 | CD184, D2S201E, FB22, HM89, HSY3RR |
| CXorf40A | 0.659 | 0.034 | endothelium and lymphocyte associated ASCH domain 1 | EOLA1, CXorf40 |
| CYTIP | 0.642 | 0.009 | cytohesin 1 interacting protein | B3-1, CASP, CYBR, CYTHIP, HE |
| DCTN4 | 0.585 | 0.019 | dynactin subunit 4 | DYN4, P62 |
| DCTN6 | 0.753 | 0.016 | dynactin subunit 6 | WS-3, WS3, p27 |
| DDIT4 | 1.384 | 0.015 | DNA damage inducible transcript 4 | Dig2, REDD-1, REDD1 |
| DIDO1 | -0.437 | 0.014 | death inducer-obliterator 1 | BYE1, C20orf158, DATF-1, DATF1, DIDO2 |
| DNAJB9 | 1.198 | 0.002 | DnaJ heat shock protein family (Hsp40) member B9 | ERdj4, MDG-1, MDG1, MST049, MSTP049 |
| DSP | -3.332 | 0.013 | desmoplakin | DCWHKTA, DP |
| DUSP22 | -0.498 | 0.033 | dual specificity phosphatase 22 | JKAP, JSP-1, JSP1, LMW-DSP2, LMWDSP2 |
| DUSP4 | 1.739 | 0.009 | dual specificity phosphatase 4 | HVH2, MKP-2, MKP2, TYP |
| DUSP6 | -1.322 | 0.002 | dual specificity phosphatase 6 | HH19, MKP3, PYST1 |
| EGR2 | 2.233 | 0.002 | early growth response 2 | AT591, CHN1, CMT1D, CMT4E, KROX20 |
| EGR3 | 2.476 | 0.008 | early growth response 3 | EGR-3, PILOT |
| EIF5 | 0.721 | 0.013 | eukaryotic translation initiation factor 5 | EIF-5, EIF-5A |
| ELF3 | 1.964 | 0.031 | E74 like ETS transcription factor 3 | EPR-1, ERT, ESE-1, ESX |
| EPGN | 2.072 | 0.033 | epithelial mitogen | ALGV3072, EPG, PRO9904 |
| ERBB2 | -1.184 | 0.008 | erb-b2 receptor tyrosine kinase 2 | CD340, HER-2, HER-2/neu, HER2, MLN 19 |
| EREG | 2.909 | 0.002 | epiregulin | EPR, ER, Ep |
| ERMN | 1.345 | 0.001 | ermin | JN, KIAA1189 |
| FAM110B | 1.424 | 0.029 | family with sequence similarity 110 member B | C8orf72 |
| FASLG | -1.269 | 0.047 | Fas ligand | ALPS1B, APT1LG1, APTL, CD178, CD95-L |
| FBXL3 | 0.625 | 0.019 | F-box and leucine rich repeat protein 3 | FBL3, FBL3A, FBXL3A, IDDSFAS |
| FBXO32 | 0.676 | 0.008 | F-box protein 32 | Fbx32, MAFbx |
| FCAR | 1.141 | 0.042 | Fc alpha receptor | CD89, CTB-61M7.2, FcalphaR, FcalphaRI |
| FEM1B | 0.458 | 0.048 | fem-1 homolog B | F1A-ALPHA, F1AA, FEM1-beta |
| FOS | 1.278 | 0.008 | Fos proto-oncogene, AP-1 transcription factor subunit | AP-1, C-FOS, p55 |
| FRS2 | 0.490 | 0.042 | fibroblast growth factor receptor substrate 2 | FRS1A, FRS2A, FRS2alpha, SNT, SNT-1 |
| G0S2 | 3.697 | 0.000 | G0/G1 switch 2 |  |
| GABARAPL1 | 1.027 | 0.002 | GABA type A receptor associated protein like 1 | APG8-LIKE, APG8L, ATG8, ATG8B, ATG8L |
| GADD45A | 1.151 | 0.002 | growth arrest and DNA damage inducible alpha | DDIT1, GADD45 |
| GFOD2 | -0.661 | 0.042 | glucose-fructose oxidoreductase domain containing 2 |  |
| GLUL | 0.713 | 0.033 | glutamate-ammonia ligase | GLNS, GS, PIG43, PIG59 |
| GNAQ | 0.549 | 0.022 | G protein subunit alpha q | CMC1, G-ALPHA-q, GAQ, SWS |
| GNLY | -0.948 | 0.021 | granulysin | D2S69E, LAG-2, LAG2, NKG5, TLA519 |
| GPR183 | 1.513 | 0.002 | G protein-coupled receptor 183 | EBI2, hEBI2 |
| GRASP | 1.395 | 0.004 | trafficking regulator and scaffold protein tamalin | TAMALIN |
| GSPT1 | 0.542 | 0.037 | G1 to S phase transition 1 | 551G9.2, ETF3A, GST1, eRF3a |
| GZF1 | 0.881 | 0.017 | GDNF inducible zinc finger protein 1 | JLSM, ZBTB23, ZNF336 |
| HBB | 7.047 | 0.001 | hemoglobin subunit beta | CD113t-C, ECYT6, beta-globin |
| HBEGF | 1.840 | 0.008 | heparin binding EGF like growth factor | DTR, DTS, DTSF, HEGFL |
| HBP1 | 0.959 | 0.001 | HMG-box transcription factor 1 |  |
| HCAR2 | 2.446 | 0.000 | hydroxycarboxylic acid receptor 2 | GPR109A, HCA2, HM74a, HM74b, NIACR1 |
| HCAR3 | 2.183 | 0.001 | hydroxycarboxylic acid receptor 3 | GPR109B, HCA3, HM74, PUMAG, Puma-g |
| HECA | 0.596 | 0.024 | hdc homolog, cell cycle regulator | HDC, HDCL, HHDC, dJ225E12.1 |
| HERPUD1 | 0.808 | 0.008 | homocysteine inducible ER protein with ubiquitin like domain 1 | HERP, Mif1, SUP |
| HIF1A | 0.846 | 0.041 | hypoxia inducible factor 1 subunit alpha | HIF-1-alpha, HIF-1A, HIF-1alpha, HIF1, HIF1-ALPHA |
| HLX | 1.069 | 0.017 | H2.0 like homeobox | HB24, HLX1 |
| IBA57 | 0.570 | 0.041 | iron-sulfur cluster assembly factor IBA57 | C1orf69, MMDS3, SPG74 |
| ID2 | 1.002 | 0.011 | inhibitor of DNA binding 2 | GIG8, ID2A, ID2H, bHLHb26 |
| IFNGR1 | 0.939 | 0.001 | interferon gamma receptor 1 | CD119, IFNGR, IMD27A, IMD27B |
| IFRD1 | 0.882 | 0.035 | interferon related developmental regulator 1 | PC4, TIS7 |
| IL10 | 2.445 | 0.005 | interleukin 10 | CSIF, GVHDS, IL-10, IL10A, TGIF |
| IL1B | 3.588 | 0.000 | interleukin 1 beta | IL-1, IL1-BETA, IL1F2, IL1beta |
| IL2RB | -0.581 | 0.046 | interleukin 2 receptor subunit beta | CD122, IL15RB, IMD63, P70-75 |
| ING3 | 0.549 | 0.040 | inhibitor of growth family member 3 | Eaf4, ING2, MEAF4, p47ING3 |
| IRS2 | 0.988 | 0.018 | insulin receptor substrate 2 | IRS-2 |
| ISCA1 | 0.484 | 0.046 | iron-sulfur cluster assembly 1 | HBLD2, ISA1, MMDS5, hIscA |
| IVNS1ABP | 0.731 | 0.018 | influenza virus NS1A binding protein | ARA3, FLARA3, HSPC068, IMD70, KLHL39 |
| JMJD6 | 0.665 | 0.015 | jumonji domain containing 6, arginine demethylase and lysine hydroxylase | PSR, PTDSR, PTDSR1 |
| JRK | -0.710 | 0.036 | Jrk helix-turn-helix protein | JH8, jerky |
| JUNB | 1.322 | 0.003 | JunB proto-oncogene, AP-1 transcription factor subunit | AP-1 |
| KANSL2 | 0.588 | 0.020 | KAT8 regulatory NSL complex subunit 2 | C12orf41, NSL2 |
| KCTD7 | -0.591 | 0.043 | potassium channel tetramerization domain containing 7 | CLN14, EPM3 |
| KLF3 | 0.454 | 0.033 | Kruppel like factor 3 | BKLF |
| KLF6 | -0.590 | 0.036 | Kruppel like factor 6 | BCD1, CBA1, COPEB, CPBP, GBF |
| LAMB3 | 2.360 | 0.019 | laminin subunit beta 3 | AI1A, BM600-125KDA, LAM5, LAMNB1 |
| LGALS9C | -1.638 | 0.046 | galectin 9C | Gal-9B, LGALS9B |
| LIMD1 | -0.542 | 0.038 | LIM domain containing 1 |  |
| LIPN | 1.751 | 0.001 | lipase family member N | ARCI8, LI4, LIPL4, bA186O14.3 |
| LRRC8C | 0.473 | 0.013 | leucine rich repeat containing 8 VRAC subunit C | AD158, FAD158 |
| LYPD3 | 1.689 | 0.029 | LY6/PLAUR domain containing 3 | C4.4A |
| MAP3K8 | 0.904 | 0.009 | mitogen-activated protein kinase kinase kinase 8 | AURA2, COT, EST, ESTF, MEKK8 |
| MASTL | 0.727 | 0.036 | microtubule associated serine/threonine kinase like | GREATWALL, GW, GWL, MAST-L, THC2 |
| MAVS | -0.422 | 0.041 | mitochondrial antiviral signaling protein | CARDIF, IPS-1, IPS1, VISA |
| MDC1 | -0.521 | 0.039 | mediator of DNA damage checkpoint 1 | NFBD1 |
| MLEC | -0.503 | 0.017 | malectin | KIAA0152 |
| MUS81 | -0.607 | 0.041 | MUS81 structure-specific endonuclease subunit | SLX3 |
| MXD1 | 1.078 | 0.002 | MAX dimerization protein 1 | BHLHC58, MAD, MAD1 |
| NABP1 | 0.776 | 0.007 | nucleic acid binding protein 1 | NABP1-OT1, OBFC2A, SOSS-B2, SSB2 |
| NAMPT | 2.301 | 0.000 | nicotinamide phosphoribosyltransferase | 1110035O14Rik, PBEF, PBEF1, VF, VISFATIN |
| NBN | 0.432 | 0.042 | nibrin | AT-V1, AT-V2, ATV, NBS, NBS1 |
| NFE2L2 | 0.894 | 0.004 | NFE2 like bZIP transcription factor 2 | HEBP1, IMDDHH, NRF2, Nrf-2 |
| NFIL3 | 1.083 | 0.012 | nuclear factor, interleukin 3 regulated | E4BP4, IL3BP1, NF-IL3A, NFIL3A |
| NFKBIZ | 0.570 | 0.023 | NFKB inhibitor zeta | IKBZ, INAP, MAIL |
| NLRP3 | 0.698 | 0.027 | NLR family pyrin domain containing 3 | AGTAVPRL, AII, AVP, C1orf7, CIAS1 |
| NMNAT1 | -0.610 | 0.048 | nicotinamide nucleotide adenylyltransferase 1 | LCA9, NMNAT, PNAT1, SHILCA |
| NMUR1 | -1.188 | 0.004 | neuromedin U receptor 1 | (FM-3), FM-3, FM3, GPC-R, GPR66 |
| NR1D2 | 0.958 | 0.005 | nuclear receptor subfamily 1 group D member 2 | BD73, EAR-1R, REVERBB, REVERBbeta, RVR |
| NR4A1 | 2.130 | 0.000 | nuclear receptor subfamily 4 group A member 1 | GFRP1, HMR, N10, NAK-1, NGFIB |
| NR4A2 | 2.456 | 0.000 | nuclear receptor subfamily 4 group A member 2 | HZF-3, NOT, NURR1, RNR1, TINUR |
| NR4A3 | 3.437 | 0.000 | nuclear receptor subfamily 4 group A member 3 | CHN, CSMF, MINOR, NOR1 |
| NUDC | -0.462 | 0.037 | nuclear distribution C, dynein complex regulator | HNUDC, MNUDC, NPD011 |
| NUDT15 | 0.774 | 0.012 | nudix hydrolase 15 | MTH2, NUDT15D |
| NUP43 | -0.716 | 0.032 | nucleoporin 43 | bA350J20.1, p42 |
| OLR1 | 3.617 | 0.002 | oxidized low density lipoprotein receptor 1 | CLEC8A, LOX1, LOXIN, SCARE1, SLOX1 |
| OSER1 | 0.606 | 0.002 | oxidative stress responsive serine rich 1 | C20orf111, HSPC207, Osr1, Perit1, dJ1183I21.1 |
| OSM | 2.608 | 0.000 | oncostatin M |  |
| OTUD1 | 0.607 | 0.010 | OTU deubiquitinase 1 | DUBA7, OTDC1 |
| PABPC4 | 0.322 | 0.043 | poly(A) binding protein cytoplasmic 4 | APP-1, APP1, PABP4, iPABP |
| PDE4B | 1.731 | 0.000 | phosphodiesterase 4B | DPDE4, PDEIVB |
| PDIK1L | 0.590 | 0.019 | PDLIM1 interacting kinase 1 like | CLIK1L, STK35L2 |
| PDP1 | 0.523 | 0.012 | pyruvate dehydrogenase phosphatase catalytic subunit 1 | PDH, PDP, PDPC, PPM2A, PPM2C |
| PELI1 | 0.803 | 0.036 | pellino E3 ubiquitin protein ligase 1 |  |
| PELI3 | -0.878 | 0.015 | pellino E3 ubiquitin protein ligase family member 3 |  |
| PER1 | 0.919 | 0.044 | period circadian regulator 1 | PER, RIGUI, hPER |
| PFKFB3 | 1.630 | 0.002 | 6-phosphofructo-2-kinase/fructose-2,6-biphosphatase 3 | IPFK2, PFK2, iPFK-2 |
| PIGM | -0.557 | 0.044 | phosphatidylinositol glycan anchor biosynthesis class M | GPI-MT-I |
| PIK3CA | 0.420 | 0.041 | phosphatidylinositol-4,5-bisphosphate 3-kinase catalytic subunit alpha | CCM4, CLAPO, CLOVE, CWS5, MCAP |
| PIK3R1 | 1.089 | 0.011 | phosphoinositide-3-kinase regulatory subunit 1 | AGM7, GRB1, IMD36, p85, p85-ALPHA |
| PLAUR | 1.218 | 0.006 | plasminogen activator, urokinase receptor | CD87, U-PAR, UPAR, URKR |
| PLK2 | 2.237 | 0.006 | polo like kinase 2 | SNK, hPlk2, hSNK |
| PLSCR1 | 0.613 | 0.034 | phospholipid scramblase 1 | MMTRA1B |
| PNPLA8 | 0.572 | 0.047 | patatin like phospholipase domain containing 8 | IPLA2-2, IPLA2G, MMLA, PNPLA-gamma, iPLA2gamma |
| PPP1R18 | -0.483 | 0.031 | protein phosphatase 1 regulatory subunit 18 | HKMT1098, KIAA1949 |
| PPP3R1 | 0.436 | 0.036 | protein phosphatase 3 regulatory subunit B, alpha | CALNB1, CNB, CNB1 |
| PTGDR | -0.917 | 0.034 | prostaglandin D2 receptor | AS1, ASRT1, DP, DP1, PTGDR1 |
| PTGER4 | 1.017 | 0.000 | prostaglandin E receptor 4 | EP4, EP4R |
| PTGS2 | 2.528 | 0.000 | prostaglandin-endoperoxide synthase 2 | COX-2, COX2, GRIPGHS, PGG/HS, PGHS-2 |
| PTP4A1 | 0.725 | 0.010 | protein tyrosine phosphatase 4A1 | HH72, PRL-1, PRL1, PTP(CAAX1), PTPCAAX1 |
| PTPRE | 0.598 | 0.048 | protein tyrosine phosphatase receptor type E | HPTPE, PTPE, R-PTP-EPSILON |
| RAB20 | 1.317 | 0.031 | RAB20, member RAS oncogene family |  |
| RASGEF1B | 1.606 | 0.002 | RasGEF domain family member 1B | GPIG4 |
| RBAK | 0.507 | 0.037 | RB associated KRAB zinc finger | ZNF769 |
| RBM44 | 1.076 | 0.033 | RNA binding motif protein 44 |  |
| RBM7 | 0.552 | 0.015 | RNA binding motif protein 7 |  |
| RGS1 | 2.122 | 0.008 | regulator of G protein signaling 1 | 1R20, BL34, HEL-S-87, IER1, IR20 |
| RGS2 | 1.868 | 0.000 | regulator of G protein signaling 2 | G0S8 |
| RGS3 | -0.896 | 0.015 | regulator of G protein signaling 3 | C2PA, RGP3 |
| RIOK3 | 0.551 | 0.044 | RIO kinase 3 | SUDD |
| RIPK2 | 1.020 | 0.002 | receptor interacting serine/threonine kinase 2 | CARD3, CARDIAK, CCK, GIG30, RICK |
| RLF | 0.532 | 0.040 | RLF zinc finger | ZN-15L, ZNF292L |
| RNASEL | -0.752 | 0.019 | ribonuclease L | PRCA1, RNS4 |
| RNF149 | 0.629 | 0.021 | ring finger protein 149 | DNAPTP2 |
| RNMT | 0.545 | 0.019 | RNA guanine-7 methyltransferase | CMT1, CMT1c, MET, Met, RG7MT1 |
| RPH3A | -1.811 | 0.035 | rabphilin 3A |  |
| SAMD8 | 0.771 | 0.034 | sterile alpha motif domain containing 8 | HEL-177, SMSr |
| SAMSN1 | 1.564 | 0.001 | SAM domain, SH3 domain and nuclear localization signals 1 | HACS1, NASH1, SASH2, SH3D6B, SLy2 |
| SAT1 | 1.088 | 0.002 | spermidine/spermine N1-acetyltransferase 1 | DC21, KFSD, KFSDX, SAT, SSAT |
| SDCBP | 0.628 | 0.038 | syndecan binding protein | MDA-9, MDA9, ST1, SYCL, TACIP18 |
| SEC14L2 | 1.586 | 0.022 | SEC14 like lipid binding 2 | C22orf6, SPF, TAP, TAP1 |
| SERPINB2 | 2.438 | 0.006 | serpin family B member 2 | HsT1201, PAI, PAI-2, PAI2, PLANH2 |
| SGK1 | 1.426 | 0.006 | serum/glucocorticoid regulated kinase 1 | SGK |
| SGTB | 0.619 | 0.043 | small glutamine rich tetratricopeptide repeat co-chaperone beta | SGT2 |
| SIK1 | 1.581 | 0.012 | salt inducible kinase 1 | DEE30, MSK, SIK, SIK-1, SIK1B |
| SKIL | 0.690 | 0.034 | SKI like proto-oncogene | SNO, SnoA, SnoI, SnoN |
| SLC16A6 | 0.809 | 0.019 | solute carrier family 16 member 6 | MCT6, MCT7 |
| SLC19A2 | 0.777 | 0.040 | solute carrier family 19 member 2 | TC1, THMD1, THT1, THTR1, TRMA |
| SLC2A3 | 1.280 | 0.012 | solute carrier family 2 member 3 | GLUT3 |
| SLC4A1 | 5.240 | 0.047 | solute carrier family 4 member 1 (Diego blood group) | AE1, BND3, CD233, CHC, DI |
| SLC5A6 | 0.769 | 0.033 | solute carrier family 5 member 6 | NERIB, SMVT |
| SMURF1 | 0.597 | 0.044 | SMAD specific E3 ubiquitin protein ligase 1 |  |
| SOCS1 | 1.598 | 0.050 | suppressor of cytokine signaling 1 | AISIMD, CIS1, CISH1, JAB, SOCS-1 |
| SOCS3 | 2.347 | 0.002 | suppressor of cytokine signaling 3 | ATOD4, CIS3, Cish3, SOCS-3, SSI-3 |
| SOD2 | 0.809 | 0.003 | superoxide dismutase 2 | GClnc1, IPO-B, IPOB, MNSOD, MVCD6 |
| SP3 | 0.568 | 0.034 | Sp3 transcription factor | SPR2 |
| SRGN | 0.662 | 0.021 | serglycin | PPG, PRG, PRG1 |
| SRSF2 | 0.553 | 0.044 | serine and arginine rich splicing factor 2 | PR264, SC-35, SC35, SFRS2, SFRS2A |
| SRSF6 | 0.409 | 0.041 | serine and arginine rich splicing factor 6 | B52, HEL-S-91, SFRS6, SRP55 |
| STIP1 | -0.498 | 0.017 | stress induced phosphoprotein 1 | HEL-S-94n, HOP, IEF-SSP-3521, P60, STI1 |
| STK17B | 0.684 | 0.018 | serine/threonine kinase 17b | DRAK2 |
| SYAP1 | 0.606 | 0.047 | synapse associated protein 1 | BSTA, PRO3113 |
| TAF1D | 0.736 | 0.044 | TATA-box binding protein associated factor, RNA polymerase I subunit D | JOSD3, RAFI41, TAF(I)41, TAFI41 |
| TBC1D15 | 0.788 | 0.008 | TBC1 domain family member 15 | RAB7-GAP |
| TEX14 | 1.866 | 0.034 | testis expressed 14, intercellular bridge forming factor | CT113, SPGF23 |
| TFRC | 0.764 | 0.007 | transferrin receptor | CD71, IMD46, T9, TFR, TFR1 |
| TGIF1 | 0.813 | 0.002 | TGFB induced factor homeobox 1 | HPE4, TGIF |
| THBS1 | 2.312 | 0.040 | thrombospondin 1 | THBS, THBS-1, TSP, TSP-1, TSP1 |
| TMED7 | 0.694 | 0.040 | transmembrane p24 trafficking protein 7 | CGI-109, p24g3, p24gamma3, p27 |
| TMEM170B | 1.004 | 0.027 | transmembrane protein 170B |  |
| TMEM70 | 0.665 | 0.040 | transmembrane protein 70 | MC5DN2 |
| TNFAIP1 | -0.687 | 0.016 | TNF alpha induced protein 1 | B12, B61, BTBD34, EDP1, hBACURD2 |
| TNFAIP6 | 2.205 | 0.015 | TNF alpha induced protein 6 | TSG-6, TSG6 |
| TP53INP2 | 1.491 | 0.009 | tumor protein p53 inducible nuclear protein 2 | C20orf110, DOR, PIG-U, PIGU, PINH |
| TRAF3IP3 | -0.586 | 0.045 | TRAF3 interacting protein 3 | T3JAM |
| TRAPPC1 | -0.644 | 0.033 | trafficking protein particle complex subunit 1 | BET5, MUM2 |
| TREM1 | 1.357 | 0.004 | triggering receptor expressed on myeloid cells 1 | CD354, TREM-1 |
| TRIM21 | -0.624 | 0.040 | tripartite motif containing 21 | RNF81, RO52, Ro/SSA, SSA, SSA1 |
| TRIM32 | -0.676 | 0.033 | tripartite motif containing 32 | BBS11, HT2A, LGMD2H, LGMDR8, TATIP |
| TTC9C | -0.725 | 0.041 | tetratricopeptide repeat domain 9C |  |
| TWISTNB | 0.475 | 0.045 | RNA polymerase I subunit F | A43, RPA43, TWISTNB |
| VAPA | 0.561 | 0.045 | VAMP associated protein A | VAMP-A, VAP-33, VAP-A, VAP33, hVAP-33 |
| VNN3 | 1.987 | 0.002 | vanin 3, pseudogene | HSA238982, VNN3 |
| YPEL5 | 0.741 | 0.023 | yippee like 5 | CGI-127 |
| YTHDF3 | 0.469 | 0.040 | YTH N6-methyladenosine RNA binding protein 3 | DF3 |
| ZBTB21 | 1.082 | 0.012 | zinc finger and BTB domain containing 21 | ZNF295 |
| ZBTB6 | 0.904 | 0.009 | zinc finger and BTB domain containing 6 | ZID, ZNF482 |
| ZC3H7A | 0.600 | 0.019 | zinc finger CCCH-type containing 7A | HSPC055, ZC3H7, ZC3HDC7 |
| ZFAND5 | 1.020 | 0.002 | zinc finger AN1-type containing 5 | ZA20D2, ZFAND5A, ZNF216 |
| ZFP3 | -0.697 | 0.036 | ZFP3 zinc finger protein | ZNF752 |
| ZFP36L2 | 0.808 | 0.022 | ZFP36 ring finger protein like 2 | BRF2, ERF-2, ERF2, RNF162C, TIS11D |
| ZNF121 | 0.513 | 0.039 | zinc finger protein 121 | D19S204, ZHC32, ZNF20 |
| ZNF14 | 1.026 | 0.026 | zinc finger protein 14 | GIOT-4, KOX6 |
| ZNF267 | 0.849 | 0.005 | zinc finger protein 267 | HZF2 |
| ZNF322 | -0.863 | 0.033 | zinc finger protein 322 | HCG12, ZNF322A, ZNF388, ZNF489 |
| ZNF331 | 1.485 | 0.007 | zinc finger protein 331 | RITA, ZNF361, ZNF463 |
| ZNF397 | -0.786 | 0.019 | zinc finger protein 397 | ZNF47, ZSCAN15 |
| ZNF514 | -0.671 | 0.038 | zinc finger protein 514 |  |
| ZNF585A | -0.754 | 0.023 | zinc finger protein 585A | Zfp27 |
| ZNF594 | -1.062 | 0.045 | zinc finger protein 594 |  |
|  |  |  |  |  |
| **IgG Positive Healthy vs Moderate** | | | | |
| ALAS2 | 6.357 | 0.023 | 5'-aminolevulinate synthase 2 | ALAS-E, ALASE, ANH1, ASB, SIDBA1 |
| ATF3 | 1.797 | 0.017 | activating transcription factor 3 |  |
| C17orf107 | 1.330 | 0.045 | chromosome 17 open reading frame 107 |  |
| CCL3 | 2.106 | 0.009 | C-C motif chemokine ligand 3 | G0S19-1, LD78ALPHA, MIP-1-alpha, MIP1A, SCYA3 |
| CD83 | 1.300 | 0.009 | CD83 molecule | BL11, HB15 |
| CXCL1 | 2.279 | 0.040 | C-X-C motif chemokine ligand 1 | FSP, GRO1, GROa, MGSA, MGSA-a |
| CXCL2 | 3.642 | 0.001 | C-X-C motif chemokine ligand 2 | CINC-2a, GRO2, GROb, MGSA-b, MIP-2a |
| CXCL3 | 4.067 | 0.002 | C-X-C motif chemokine ligand 3 | CINC-2b, GRO3, GROg, MIP-2b, MIP2B |
| CXCL8 | 4.768 | 0.000 | C-X-C motif chemokine ligand 8 | GCP-1, GCP1, IL8, LECT, LUCT |
| DSP | -3.226 | 0.045 | desmoplakin | DCWHKTA, DP |
| DUSP6 | -1.401 | 0.003 | dual specificity phosphatase 6 | HH19, MKP3, PYST1 |
| EGR2 | 1.660 | 0.045 | early growth response 2 | AT591, CHN1, CMT1D, CMT4E, KROX20 |
| EREG | 2.059 | 0.048 | epiregulin | EPR, ER, Ep |
| FOS | 1.719 | 0.002 | Fos proto-oncogene, AP-1 transcription factor subunit | AP-1, C-FOS, p55 |
| G0S2 | 3.475 | 0.003 | G0/G1 switch 2 |  |
| HBB | 7.207 | 0.004 | hemoglobin subunit beta | CD113t-C, ECYT6, beta-globin |
| HBEGF | 1.800 | 0.029 | heparin binding EGF like growth factor | DTR, DTS, DTSF, HEGFL |
| HCAR2 | 2.219 | 0.003 | hydroxycarboxylic acid receptor 2 | GPR109A, HCA2, HM74a, HM74b, NIACR1 |
| HCAR3 | 1.876 | 0.018 | hydroxycarboxylic acid receptor 3 | GPR109B, HCA3, HM74, PUMAG, Puma-g |
| IL10 | 2.676 | 0.009 | interleukin 10 | CSIF, GVHDS, IL-10, IL10A, TGIF |
| IL1B | 2.699 | 0.001 | interleukin 1 beta | IL-1, IL1-BETA, IL1F2, IL1beta |
| JUNB | 1.136 | 0.029 | JunB proto-oncogene, AP-1 transcription factor subunit | AP-1 |
| LIPN | 1.446 | 0.020 | lipase family member N | ARCI8, LI4, LIPL4, bA186O14.3 |
| MXD1 | 0.882 | 0.023 | MAX dimerization protein 1 | BHLHC58, MAD, MAD1 |
| NABP1 | 0.692 | 0.048 | nucleic acid binding protein 1 | NABP1-OT1, OBFC2A, SOSS-B2, SSB2 |
| NAMPT | 1.623 | 0.009 | nicotinamide phosphoribosyltransferase | 1110035O14Rik, PBEF, PBEF1, VF, VISFATIN |
| NR4A1 | 1.490 | 0.018 | nuclear receptor subfamily 4 group A member 1 | GFRP1, HMR, N10, NAK-1, NGFIB |
| NR4A2 | 1.909 | 0.005 | nuclear receptor subfamily 4 group A member 2 | HZF-3, NOT, NURR1, RNR1, TINUR |
| NR4A3 | 2.282 | 0.014 | nuclear receptor subfamily 4 group A member 3 | CHN, CSMF, MINOR, NOR1 |
| OLR1 | 3.184 | 0.023 | oxidized low density lipoprotein receptor 1 | CLEC8A, LOX1, LOXIN, SCARE1, SLOX1 |
| OSM | 1.850 | 0.001 | oncostatin M |  |
| PDE4B | 1.016 | 0.040 | phosphodiesterase 4B | DPDE4, PDEIVB |
| PLK2 | 2.325 | 0.014 | polo like kinase 2 | SNK, hPlk2, hSNK |
| PTGER4 | 0.827 | 0.003 | prostaglandin E receptor 4 | EP4, EP4R |
| PTGS2 | 1.766 | 0.006 | prostaglandin-endoperoxide synthase 2 | COX-2, COX2, GRIPGHS, PGG/HS, PGHS-2 |
| RGS2 | 1.585 | 0.000 | regulator of G protein signaling 2 | G0S8 |
| RIPK2 | 0.905 | 0.018 | receptor interacting serine/threonine kinase 2 | CARD3, CARDIAK, CCK, GIG30, RICK |
| SERPINB2 | 2.835 | 0.010 | serpin family B member 2 | HsT1201, PAI, PAI-2, PAI2, PLANH2 |
| SOCS3 | 1.973 | 0.016 | suppressor of cytokine signaling 3 | ATOD4, CIS3, Cish3, SOCS-3, SSI-3 |
| SOD2 | 0.661 | 0.048 | superoxide dismutase 2 | GClnc1, IPO-B, IPOB, MNSOD, MVCD6 |
| TGIF1 | 0.640 | 0.046 | TGFB induced factor homeobox 1 | HPE4, TGIF |
| TREM1 | 1.248 | 0.023 | triggering receptor expressed on myeloid cells 1 | CD354, TREM-1 |
| ZFAND5 | 0.793 | 0.045 | zinc finger AN1-type containing 5 | ZA20D2, ZFAND5A, ZNF216 |
|  |  |  |  |  |

**Supplemental Data Table 4**. Genes for transcription factors inferred from accessible chromatin using (A) footprint depth or (B) average per-cell gene expression (B) in IgG- COVID-19 subjects with mild or moderate symptoms.

| **A** |  |  |  |  |  |  |
| --- | --- | --- | --- | --- | --- | --- |
| **Gene Symbol** | **Gene Name** | **Alias** | **FA** | **FD** | **Dist From Mean. FD** |  |
| NFYC | nuclear transcription factor Y subunit gamma | CBF-C, CBFC, H1TF2A, HAP5, HSM | 1.776889 | 0.158786 | 1.382164 |  |
| NFYC | nuclear transcription factor Y subunit gamma | CBF-C, CBFC, H1TF2A, HAP5, HSM | 1.776889 | 0.158786 | 1.382164 |  |
| CREB3L4 | cAMP responsive element binding protein 3 like 4 | AIBZIP, ATCE1, CREB3, CREB4, JAL | 1.885224 | 0.117258 | 1.487107 |  |
| ETV3 | ETS variant transcription factor 3 | METS, PE-1, PE1 | 1.57684 | 0.851595 | 1.433826 |  |
| ELK4 | ETS transcription factor ELK4 | SAP1 | 1.423513 | 0.862084 | 1.317563 |  |
| ATF3 | activating transcription factor 3 |  | 1.546082 | 0.36752 | 1.193719 |  |
| BATF3 | basic leucine zipper ATF-like transcription factor 3 | JDP1, JUNDM1, SNFT | 1.661989 | 0.06807 | 1.261931 |  |
| CREM | cAMP responsive element modulator | CREM-2, ICER, hCREM-2 | 1.576554 | 0.096096 | 1.177735 |  |
| ARNTL | aryl hydrocarbon receptor nuclear translocator like | BMAL1, BMAL1c, JAP3, MOP3, PASD3 | 1.514937 | 0.366141 | 1.163467 |  |
| ELF5 | E74 like ETS transcription factor 5 | ESE2 | 1.441917 | 0.573613 | 1.173916 |  |
| EHF | ETS homologous factor | ESE3, ESE3B, ESEJ | 1.388274 | 0.637534 | 1.158708 |  |
| ETS1 | ETS proto-oncogene 1, transcription factor | ETS-1, EWSR2, c-ets-1, p54 | 1.584609 | 0.979662 | 1.516775 |  |
| FLI1 | Fli-1 proto-oncogene, ETS transcription factor | BDPLT21, EWSR2, FLI-1, SIC-1 | 1.588106 | 1.012869 | 1.540442 |  |
| ATF1 | activating transcription factor 1 | EWS-ATF1, FUS/ATF-1, TREB36 | 1.879582 | 0.226322 | 1.491753 |  |
| ATF7 | activating transcription factor 7 | ATFA | 1.934297 | 0.118486 | 1.53617 |  |
| ELK3 | ETS transcription factor ELK3 | ERP, NET, SAP-2, SAP2 | 1.538186 | 1.039345 | 1.51972 |  |
| NFYB | nuclear transcription factor Y subunit beta | CBF-A, CBF-B, HAP3, NF-YB | 1.948674 | 0.077309 | 1.548764 |  |
| E4F1 | E4F transcription factor 1 | E4F | 1.826142 | 0.28039 | 1.447094 |  |
| CTCF | CCCTC-binding factor | CFAP108, FAP108, MRD21 | 1.616338 | 0.085294 | 1.216937 |  |
| ETV4 | ETS variant transcription factor 4 | E1A-F, E1AF, PEA3, PEAS3 | 1.527958 | 1.017851 | 1.49784 |  |
| CREB3L3 | cAMP responsive element binding protein 3 like 3 | CREB-H, CREBH, HYST1481, HYTG2 | 1.885224 | 0.117258 | 1.487107 |  |
| DNMT1 | DNA methyltransferase 1 | ADCADN, AIM, CXXC9, DNMT, HSN1E | 0.166182 | 1.364419 | 1.353204 |  |
| ETV2 | ETS variant transcription factor 2 | ER71, ETSRP71 | 1.547392 | 0.673181 | 1.314042 |  |
| ERF | ETS2 repressor factor | CHYTS, CRS4, PE-2, PE2 | 1.624718 | 0.819223 | 1.455592 |  |
| SPIB | Spi-B transcription factor | SPI-B | 1.44419 | 0.513438 | 1.149441 |  |
| CEBPZ | CCAAT enhancer binding protein zeta | CBF, CBF2, HSP-CBF, NOC1 | 1.781448 | 0.062038 | 1.3812 |  |
| ATF2 | activating transcription factor 2 | CRE-BP1, CREB-2, CREB2, HB16, TREB7 | 1.57009 | 0.144847 | 1.174971 |  |
| CREB1 | cAMP responsive element binding protein 1 | CREB, CREB-1 | 1.954375 | 0.251782 | 1.569305 |  |
| FEV | FEV transcription factor, ETS family member | HSRNAFEV, PET-1 | 1.618143 | 0.915124 | 1.504308 |  |
| TCFL5 | transcription factor like 5 | CHA, E2BP-1, Figlb, SOSF1, bHLHe82 | 0.725189 | 1.206129 | 1.218491 |  |
| GABPA | GA binding protein transcription factor subunit alpha | E4TF1-60, E4TF1A, NFT2, NRF2, NRF2A | 1.442925 | 0.887873 | 1.34891 |  |
| ERG | ETS transcription factor ERG | erg-3, p55 | 1.623005 | 0.937047 | 1.521191 |  |
| ETV5 | ETS variant transcription factor 5 | ERM | 1.50067 | 1.045481 | 1.495996 |  |
| NFYA | nuclear transcription factor Y subunit alpha | CBF-A, CBF-B, HAP2, NF-YA | 1.801068 | 0.240936 | 1.416033 |  |
| ETV1 | ETS variant transcription factor 1 | ER81 | 1.531165 | 1.011053 | 1.495796 |  |
| CREB5 | cAMP responsive element binding protein 5 | CRE-BPA, CREB-5, CREBPA | 1.924359 | 0.115324 | 1.526072 |  |
| NRF1 | nuclear respiratory factor 1 | ALPHA-PAL | 1.564161 | 1.22023 | 1.663306 |  |
| CREB3 | cAMP responsive element binding protein 3 | LUMAN, LZIP, sLZIP | 1.885224 | 0.117258 | 1.487107 |  |
| PBX3 | PBX homeobox 3 |  | 1.859968 | 0.041833 | 1.459421 |  |
| ELK1 | ETS transcription factor ELK1 |  | 1.524453 | 1.019351 | 1.496193 |  |
| ELF4 | E74 like ETS transcription factor 4 | ELFR, MEF | 1.393348 | 0.692196 | 1.192425 |  |
|  |  |  |  |  |  |  |
| **B** |  |  |  |  |  |  |
| **Gene Symbol** | **Gene Name** | **Alias** | **FA** | **FD** | **GEX** | **Dist From Mean GEX** |
| ENO1 | enolase 1 | ENO1L1, HEL-S-17, MPB1, NNE, PPH | 0.480553 | 0.029711 | 2.186137 | 2.045101 |
| NFYC | nuclear transcription factor Y subunit gamma | CBF-C, CBFC, H1TF2A, HAP5, HSM | 1.776889 | 0.158786 | 0.440122 | 1.408098 |
| NFYC | nuclear transcription factor Y subunit gamma | CBF-C, CBFC, H1TF2A, HAP5, HSM | 1.776889 | 0.158786 | 0.028528 | 1.381026 |
| YBX1 | Y-box binding protein 1 | BP-8, CBF-A, CSDA2, CSDB, DBPB | 1.218425 | 0.234696 | 2.86644 | 2.843971 |
| JUN | Jun proto-oncogene, AP-1 transcription factor subunit | AP-1, AP1, c-Jun, cJUN, p39 | 1.465519 | -0.16318 | 1.648559 | 1.844452 |
| CREB3L4 | cAMP responsive element binding protein 3 like 4 | AIBZIP, ATCE1, CREB3, CREB4, JAL | 1.885224 | 0.117258 | 0.05561 | 1.487188 |
| ETV3 | ETS variant transcription factor 3 | METS, PE-1, PE1 | 1.57684 | 0.851595 | 0.125082 | 1.176388 |
| ATF3 | activating transcription factor 3 |  | 1.546082 | 0.36752 | 0.130201 | 1.145567 |
| BATF3 | basic leucine zipper ATF-like transcription factor 3 | JDP1, JUNDM1, SNFT | 1.661989 | 0.06807 | 0.105881 | 1.261941 |
| KLF6 | Kruppel like factor 6 | BCD1, CBA1, COPEB, CPBP, GBF | 0.647414 | 0.295195 | 2.284001 | 2.155579 |
| CREM | cAMP responsive element modulator | CREM-2, ICER, hCREM-2 | 1.576554 | 0.096096 | 0.247401 | 1.180632 |
| SPI1 | Spi-1 proto-oncogene | AGM10, OF, PU.1, SFPI1, SPI-1 | 0.9168 | 0.089468 | 1.252244 | 1.223843 |
| ETS1 | ETS proto-oncogene 1, transcription factor | ETS-1, EWSR2, c-ets-1, p54 | 1.584609 | 0.979662 | 0.975752 | 1.447778 |
| FLI1 | Fli-1 proto-oncogene, ETS transcription factor | BDPLT21, EWSR2, FLI-1, SIC-1 | 1.588106 | 1.012869 | 0.908123 | 1.412883 |
| ATF1 | activating transcription factor 1 | EWS-ATF1, FUS/ATF-1, TREB36 | 1.879582 | 0.226322 | 0.269237 | 1.484411 |
| ATF7 | activating transcription factor 7 | ATFA | 1.934297 | 0.118486 | 0.132508 | 1.533748 |
| ELK3 | ETS transcription factor ELK3 | ERP, NET, SAP-2, SAP2 | 1.538186 | 1.039345 | 0.253671 | 1.143013 |
| NFYB | nuclear transcription factor Y subunit beta | CBF-A, CBF-B, HAP3, NF-YB | 1.948674 | 0.077309 | 0.121707 | 1.548232 |
| ELF1 | E74 like ETS transcription factor 1 | EFTUD1, RIA1 | 1.292026 | 0.750574 | 1.18418 | 1.370971 |
| FOS | Fos proto-oncogene, AP-1 transcription factor subunit | AP-1, C-FOS, p55 | 1.466687 | -0.16769 | 2.461686 | 2.552399 |
| E4F1 | E4F transcription factor 1 | E4F | 1.826142 | 0.28039 | 0.198815 | 1.426667 |
| CTCF | CCCTC-binding factor | CFAP108, FAP108, MRD21 | 1.616338 | 0.085294 | 0.315785 | 1.228028 |
| MLX | MAX dimerization protein MLX | MAD7, MXD7, TCFL4, TF4, bHLHd13 | 1.485843 | 0.309495 | 0.569822 | 1.166323 |
| CREB3L3 | cAMP responsive element binding protein 3 like 3 | CREB-H, CREBH, HYST1481, HYTG2 | 1.885224 | 0.117258 | 2.10E-06 | 1.491474 |
| JUNB | JunB proto-oncogene, AP-1 transcription factor subunit | AP-1 | 1.516623 | -0.21453 | 3.399148 | 3.442477 |
| KLF2 | Kruppel like factor 2 | LKLF | 1.084616 | 0.074151 | 1.92472 | 1.908888 |
| JUND | JunD proto-oncogene, AP-1 transcription factor subunit | AP-1 | 1.442277 | -0.20765 | 0.734056 | 1.197893 |
| ETV2 | ETS variant transcription factor 2 | ER71, ETSRP71 | 1.547392 | 0.673181 | 0.036345 | 1.151721 |
| ERF | ETS2 repressor factor | CHYTS, CRS4, PE-2, PE2 | 1.624718 | 0.819223 | 0.062532 | 1.226752 |
| CEBPZ | CCAAT enhancer binding protein zeta | CBF, CBF2, HSP-CBF, NOC1 | 1.781448 | 0.062038 | 0.494458 | 1.424989 |
| ATF2 | activating transcription factor 2 | CRE-BP1, CREB-2, CREB2, HB16, TREB7 | 1.57009 | 0.144847 | 0.207847 | 1.171326 |
| CREB1 | cAMP responsive element binding protein 1 | CREB, CREB-1 | 1.954375 | 0.251782 | 0.436478 | 1.58134 |
| FEV | FEV transcription factor, ETS family member | HSRNAFEV, PET-1 | 1.618143 | 0.915124 | 0 | 1.225883 |
| ERG | ETS transcription factor ERG | erg-3, p55 | 1.623005 | 0.937047 | 8.33E-05 | 1.230702 |
| IRF1 | interferon regulatory factor 1 | IRF-1, MAR | 0.698679 | 0.194302 | 1.784493 | 1.668735 |
| NFYA | nuclear transcription factor Y subunit alpha | CBF-A, CBF-B, HAP2, NF-YA | 1.801068 | 0.240936 | 0.074494 | 1.40214 |
| CREB5 | cAMP responsive element binding protein 5 | CRE-BPA, CREB-5, CREBPA | 1.924359 | 0.115324 | 0.147318 | 1.523784 |
| IKZF1 | IKAROS family zinc finger 1 | CVID13, Hs.54452, IK1, IKAROS, LYF1 | -0.06456 | 0.043535 | 1.215927 | 1.16978 |
| NRF1 | nuclear respiratory factor 1 | ALPHA-PAL | 1.564161 | 1.22023 | 0.150816 | 1.163608 |
| CREB3 | cAMP responsive element binding protein 3 | LUMAN, LZIP, sLZIP | 1.885224 | 0.117258 | 0.247888 | 1.48837 |
| PBX3 | PBX homeobox 3 |  | 1.859968 | 0.041833 | 0.085855 | 1.460489 |

Supplemental Methods

Cohort recruitment and biological sample collection

Protection of human subjects was in accordance with research protocols approved by the Duke University Institutional Review Board, consistent with the Declaration of Helsinki. Written informed consent was obtained from all research subjects or their legally authorized representatives. Subjects with confirmed or suspected SARS-CoV-2 infection or their close contacts were identified in the outpatient setting and enrolled into the Molecular and Epidemiological Study of Suspected Infection protocol (MESSI, IRB Pro00100241). All close contacts and subjects with mild or moderate COVID-19 were longitudinally sampled from enrollment to convalescent phase. Biological samples were collected prospectively at first visit (Day 0) and at weekly intervals on Day 7 and Day 14. At each visit, infection with SARS-CoV-2 was confirmed using qPCR on nasopharyngeal (NP) swab samples, and serology testing was performed for IgG against the SARS-CoV-2 spike domain (Supplemental Tables 1 and 2). All subjects with mild or moderate COVID-19 progressed from seronegative (IgG-) to seropositive (IgG+). Close contacts were PCR negative and IgG- at all time points; healthy controls were enrolled pre-pandemic and were not tested for SARS-CoV-2 or spike protein IgG. Self-reported symptom surveys were performed at each visit. To categorize symptom severity, the sum of 38 defined symptom categories, each scored 0-4 (0-none, 1-mild, 2-moderate, 3-severe, 4-very severe), was determined from symptom onset through each longitudinal collection.

SARS-CoV-2 IgG ELISA

Antibody response testing was performed using the anti-SARS-CoV-2 spike S1 domain IgG ELISA assay (EUROIMMUN Medizinische Labordiagnostika AG, Lübeck, Germany) according to manufacturer’s instructions. Test results were evaluated by calculating the ratio of the optical density (OD) of the test sample over the OD of the calibrator sample where a ratio of <0.8 was interpreted as negative and ratio of 1.1 or greater as positive; a ratio of 0.8 to <1.1 was considered indeterminate and not used in this study.

SARS-CoV-2 quantification by qRT-PCR

Nasal swab Viral Transport Medium (VTM) was aliquoted and cryopreserved from study subjects to determine SARS-CoV-2 N1 gene copy number by RT-PCR to stratify subjects as COVID PCR positive or negative. Viral RNA was extracted from 140 uL of VTM according to manufacturer’s instructions (QiaAmp Viral RNA minikit). SARS-CoV-2 nucleocapsid (N1) and human RNase P (RPP30) RNA copies were determined using 5 µL of isolated RNA in the CDC-designed kit (CDC-006-00019, Revision: 03, Integrated DNA Technologies 2019-nCoV kit). Standard quantitative RT-PCR (TaqPath 1-step RT qPCR Master Mix, Thermofisher) was run with test RNA and gene-specific standard curves (2e5 copy/mL – 20 copy/mL). Regression analysis was used to determine gene copy number and corrected to report copies/mL of VTM. Samples with a Ct value less than 35 were scored as COVID PCR negative and samples greater than or equal to 35 were scored COVID PCR positive.

Purification of PBMCs

PBMCs were purified using the Ficoll-Hypaque density gradient method according to manufacturer’s instructions. Briefly, whole blood was collected in ACD Vacutainer tubes (BD) and processed within 8 hours by dilution 1:2 in PBS, layered onto the Ficoll-Hypaque (Sigma Aldrich) in 50 ml conical tubes, and centrifuged at 420 x g for 25 minutes. Buffy coat was collected, washed twice in D-PBS by centrifugation at 400 x g for 10 minutes to isolate peripheral blood mononuclear cells (PBMCs) which were assessed for viability and cell count using a Vi-Cell automated cell counter (Beckman-Coulter). PBMCs were adjusted to 10x106 cells/ml in cryopreservation media (90% FBS, 10% DMSO), frozen at -80oC using CoolCell LX (BioCision) for 12-24 hours and stored in liquid nitrogen vapor phase.

RNA extraction, total RNA-seq, and data processing

RNA was extracted from 300K cells using the Zymo Direct-zol miniprep kit (Cat# R2051) and RNA quality assessed using the Agilent DNA tape screen assay. The RNA Integrity Number (RIN) scores for all samples were > 7.0. Total RNA libraries were generated using the NuGEN Ovation® SoLo RNA-Seq Library Preparation Kit (Cat# 0500-96). Libraries were sequenced using Illumina NovaSeq 6000 instrument with S4 flow cell and 150 base pair paired-end reads. FASTQ files were generated from the NovaSeq BCL outputs and quality was assessed with FASTQC^1^. Eukaryotic rRNA sequences were removed using SortMeRNA, and the remaining reads were aligned against the hg19 human reference genome using STAR and the following commands: STAR –genomeDir /path/to/STARIndex/ --sjdbGTFfile /path/to/gene.gtf –readFilesIn /path/to/R1.fastq /path/toR2.fastq –runThreadN 8 –twopassMode Basic –outWigType bedGraph –outSAMtype BAM SortedByCoordinate –readFilesCommand zcat –outReadsUnmapped Fastx –outFileNamePrefix $sampleID. Following alignment, the gene count matrix was generated using featureCounts^2,3^.

Differential gene expression analysis

Differentially expressed genes were identified between subjects with different disease severity using the limma package and voom to model variance^4,5^. Subject sex and RNA-seq library batch were added as variables to the design formula to account for expected technical variation in the counts. Genes not annotated as protein coding in the Ensembl hg19 assembly were filtered from the gene count matrix. False discovery rate adjustment was performed for the p-values using the Benjamini-Hochberg procedure. A gene was defined as significantly differentially expressed if the adjusted p-value <= 0.05. Results from limma were passed to the EnhancedVolcano package to generate volcano plots with the same p-value threshold^6^. Two iterations of duplicate correlation and variance modeling implemented in voom were used to estimate the proportion of gene count variance associated with each experimental variable^6^.

Nuclei purification, ATAC-seq and data processing

Nuclei were extracted from frozen PBMCs. Briefly, 100K cells were spun down at 300 x g for 5 minutes at 4^o^C. The supernatant was removed, and cells were mixed with 100 uL of lysis buffer (10mM NaCl, 3mM MgCl2, 10mM Tris-HCl pH7.4, 0.1% Tween-20, 0.1% Nonidet^TM^ P40) and lysed on ice for 4 minutes. Wash buffer (1 mL; 10mM NaCl, 3mM MgCl2, 10mM Tris-HCl pH7.4, 0.1% Tween20) was added before centrifuging at 500 x g for 5 minutes at 4^o^C. ATAC-seq libraries were generated as presented earlier^7^. Briefly, transposition mix (25 μL 2× TD buffer, 2.5 μL transposase (Tn5, 100 nM final), 22.5 μL water) (Illumina Cat# 20031198) was added to the nuclear pellets, incubated at 37 °C for 30 minutes, and samples purified using the Qiagen MinElute PCR Purification Kit (Qiagen Cat#28004). DNA fragments were PCR amplified for a total of 10-11 cycles and resulting libraries purified using the Qiagen MinElute PCR Purification Kit. The libraries were sequenced with an Illumina Novaseq 6000 S4 flow cell using 100 bp paired-end reads. FASTQ files were generated from the NovaSeq BCL outputs and used as input to the ENCODE ATAC-seq pipeline (<https://github.com/ENCODE-DCC/atac-seq-pipeline>) using the MACS2 peak-caller with all default parameters. Output narrowPeak files and aligned BAM files were used for downstream analysis.

Differential chromatin accessibility analysis

Differential accessibility was calculated between groups of subjects with different disease severity using the csaw package^8^. The aligned BAM files were used as input for analysis performed with csaw. First, aligned reads were counted in genome-wide windows of 10 bp, filtering out windows with fewer than 30 reads. Windows were then extended by 2 kb on either size of the window center, and these regions were used to calculate coverage in window flanking regions. Windows with less than 3 fold-change local enrichment were filtered out and not used for statistical modeling. Non-linear normalization was performed with the normOffsets() function. Differential testing was then applied to the window counts using a quali-likelihood negative binomial generalized log-linear model for the relevant experimental contrasts. Differentially accessible windows were defined as having a p-value <= 0.05. Windows were split into three groups: differentially accessible windows (1) only observed in mild subjects; (2) only observed in moderate subjects; and (3) observed in both mild and moderate subjects. These groups of windows were annotated to genomic features using ChIPseeker and used as input to gprofiler2 to perform functional enrichment analysis via the g:Profiler web tool^9^. Gene lists were used to identify enrichment in the TRANSFAC (TF) database, REACTOME (REAC) database, and the molecular function, biological processes, and cellular component gene ontologies (GO: MF, GO: BP, GO: CC).

Single-cell (sc)RNA-seq and data processing

Frozen PBMCs were thawed, and count and cell viability were measured by Countess II. The cell viability exceeded 80% for all samples except PBMC samples from CC subjects, which had viability between 70-80%. For single cell (sc) RNA-seq, 200K cells were aliquoted, spun down, resuspended in 30 uL PBS+0.04%BSA+0.2U/uL RNase inhibitor, and counted using Countess II. GEM generation, post GEMRT cleanup, cDNA amplification, and library construction were performed following 10X Genomics Single Cell 5’ v1 chemistry and quality was assessed using Agilent DNA tape screen assay. Libraries were then pooled and sequenced using Illumina NovaSeq 6000 platform with the goal of reaching saturation or 20,000 unique reads per cell on average. Sequencing data were used as input to the 10x Genomics Cell Ranger pipeline to demultiplex BCL files, generate FASTQs, and generate feature counts for each library. For dimensionality reduction and cell type annotation, gene-barcode matrices generated using CellRanger count were analyzed using Seurat 3 with the default parameters unless otherwise specified^10^. Cells with > 5% of reads mapping to the mitochondrial genome or > 2500 genes detected were removed from the analysis. Counts were log-normalized, and the top 2000 variable features were identified. Principal component analysis was performed using these variable genes, and the top 20 principal components were used for downstream analysis. UMAP dimensionality reduction was performed using the top 20 principal components identified using the Harmony package^11^. Graph-based clustering was performed with resolution = 0.5. Cell types were inferred by using the DatabaseImmuneCellExpressionData() function from the SingleR package^12^. Labels were confirmed by identification of differentially expressed genes using the FindAllMarkers() function from Seurat^10^.

For regulatory network inference, the scRNA-seq Seurat object was converted into a SingleCellExperiment and used as input to analysis with the SCENIC package^13^. Cells from seronegative MS and PS subjects were re-clustered using Monocle 3, and the top 100 marker genes were computed for each cell partition^14^. The standard workflow for running the SCENIC analysis was then performed using the count matrix for these marker genes as input (<https://github.com/aertslab/SCENIC>). Briefly, GENIE3 was used to identify regulons of transcription factors and their downstream regulatory targets with correlated co-expression, and AUCell was then used to score the activity of these regulons in each cluster. The ‘top10perTarget’ co-expression parameter value was used to prune the list of scored regulons.

Single-cell (sc)ATAC-seq, data processing and analysis

PBMCs were thawed and nuclei were extracted as for ATAC-seq. The single-cell suspensions of scATAC-seq samples were converted to barcoded scATAC-seq libraries using the Chromium Single Cell 5′ Library, Gel Bead and Multiplex Kit, and Chip Kit (10x Genomics). The Chromium Single Cell 5′ v2 Reagent (10x Genomics, 120237) kit was used to prepare single-cell ATAC libraries according to the manufacturer’s instructions. Quality was assessed using Agilent DNA tape screen assay. Libraries were then pooled and sequenced using Illumina NovaSeq platform with the goal of reaching saturation or 25,000 unique reads per nuclei on average. Sequencing data were used as input to the 10x Genomics Cell Ranger ATAC pipeline to demultiplex BCL files, generate FASTQs, and generate feature counts for each library.

For scRNA-seq and scATAC-seq integration, fragment file outputs generated using CellRanger ATAC count were analyzed using ArchR following the standard workflow and with default parameters unless otherwise specified^15^. Cells with a transcription start site enrichment score < 4, cells with fewer than 1000 detected fragments, and putative doublets were removed from downstream analysis. Dimensionality reduction was computed using iterative latent semantic indexing (LSI), and batch effect correction was applied using Harmony. Graph-based clustering was performed using the FindClusters() method from Seurat 3 with resolution = 0.8. UMAP embeddings were calculated with the top 30 principal components from either LSI or Harmony. Constrained integration was performed using the addGeneIntegrationMatrix() method and scRNA-seq cell type annotations were used to label the identify of scATAC-seq clusters. Pseudo-scRNA-seq gene expression profiles were estimated for each cell type using the addGeneIntegrationMatrix() function from ArchR during the constrained label transfer procedure.

For feature and motif enrichment analysis, peak calling was performed using MACS2 via the addReproduciblePeakSet() method in ArchR which uses pseudo-bulk replicates of cells grouped on a specific design variable. Differentially accessible peaks were identified between two groups and visualized using the ArchR methods getMarkerFeatures() and markerPlot(), respectively. Significance was defined as FDR <= 0.1 and absolute log2 fold change >= 0.5 unless otherwise specified. The ‘cisbp’ motif set was imported from TFBSTools using the ArchR addMotifAnnotations() method, and motif enrichment in differentially accessible peaks was performed using the peakAnnoEnrichment() method. Accessibility estimates were compared to the average gene expression over all cells for the genes that encode each transcription factor. The gene integration matrix was constructed using the .getGroupMatrix() function from ArchR and specifying which subject cohorts to compare.

For integrative analysis with scRNA-seq, the correlations between chromVAR transcription factor deviation scores and gene expression data were calculated using the ArchR method correlateMatrices() to identify activators and repressors. Peak-to-gene linkages were calculated using the addPeak2GeneLinks() method in ArchR using a correlation cutoff of 0.5 and resolution = 1. This approach uses low-overlapping cell aggregates to reduce noise that arises from doing correlative analyses with sparse scATAC-seq datasets. Peak-to-gene linkages were plotted against peak accessibility at gene bodies within DORCs for each cell type. DORC genes were defined as gene loci with > 10 peak-to-gene linkages, and these sites were used as input to the web tool Seanalysis to identify regulation by a known super-enhancer in peripheral blood cells^16,17^. The activity of the top-ranked transcription factor regulators that were correlated with scRNA-seq clusters was estimated for each scATAC-seq cluster. These activities were used to calculate Pearson correlation coefficients between scATAC-seq clusters (C1-C8) and scRNA-seq clusters (C1-C11) were calculated to identify scATAC-seq clusters with similar regulatory network activity.

Topic-based clustering was performed for the CD14+ monocytes from the mild or moderate subject cohorts using the R package cisTopic^18^. The cisTopic object was constructed from the CellRanger ATAC outputs for each sample and subset to include only cells annotated as CD14+ monocytes following constrained integration with ArchR, as previously described. An optimized model with 30 topics was selected using the runCGSModels() function from cisTopic and maximizing the log-likelihood. Dimensionality reduction was performed using the UMAP algorithm and cells were clustered using the R package densityClust with parameter values rho = 50 and delta = 2.5. Cells were colored by either cluster or subject disease severity.

**References**

1 Andrews, S. *FastQC: A Quality Control Tool for High Throughput Sequence Data [Online]*, <<https://www.bioinformatics.babraham.ac.uk/projects/fastqc/>> (2015).

2 Dobin, A. *et al.* STAR: ultrafast universal RNA-seq aligner. *Bioinformatics* **29**, 15-21, doi:10.1093/bioinformatics/bts635 (2013).

3 Kopylova, E., Noé, L. & Touzet, H. SortMeRNA: fast and accurate filtering of ribosomal RNAs in metatranscriptomic data. *Bioinformatics* **28**, 3211-3217, doi:10.1093/bioinformatics/bts611 (2012).

4 Ritchie, M. E. *et al.* limma powers differential expression analyses for RNA-sequencing and microarray studies. *Nucleic Acids Res* **43**, e47, doi:10.1093/nar/gkv007 (2015).

5 Law, C. W., Chen, Y., Shi, W. & Smyth, G. K. voom: Precision weights unlock linear model analysis tools for RNA-seq read counts. *Genome Biol* **15**, R29, doi:10.1186/gb-2014-15-2-r29 (2014).

6 EnhancedVolcano: Publication-ready volcano plots with enhanced colouring and labeling. (R Package, 2021).

7 Buenrostro, J. D., Wu, B., Chang, H. Y. & Greenleaf, W. J. ATAC-seq: A Method for Assaying Chromatin Accessibility Genome-Wide. *Curr Protoc Mol Biol* **109**, 21 29 21-21 29 29, doi:10.1002/0471142727.mb2129s109 (2015).

8 Lun, A. T. & Smyth, G. K. csaw: a Bioconductor package for differential binding analysis of ChIP-seq data using sliding windows. *Nucleic Acids Res* **44**, e45, doi:10.1093/nar/gkv1191 (2016).

9 Raudvere, U. *et al.* g:Profiler: a web server for functional enrichment analysis and conversions of gene lists (2019 update). *Nucleic Acids Research* **47**, W191-W198, doi:10.1093/nar/gkz369 (2019).

10 Stuart, T. *et al.* Comprehensive Integration of Single-Cell Data. *Cell* **177**, 1888-1902.e1821, doi:10.1016/j.cell.2019.05.031 (2019).

11 Korsunsky, I. *et al.* Fast, sensitive and accurate integration of single-cell data with Harmony. *Nature Methods* **16**, 1289-1296, doi:10.1038/s41592-019-0619-0 (2019).

12 Aran, D. *et al.* Reference-based analysis of lung single-cell sequencing reveals a transitional profibrotic macrophage. *Nature Immunology* **20**, 163-172, doi:10.1038/s41590-018-0276-y (2019).

13 Aibar, S. *et al.* SCENIC: single-cell regulatory network inference and clustering. *Nature Methods* **14**, 1083-1086, doi:10.1038/nmeth.4463 (2017).

14 Trapnell, C. *et al.* The dynamics and regulators of cell fate decisions are revealed by pseudotemporal ordering of single cells. *Nature Biotechnology* **32**, 381-386, doi:10.1038/nbt.2859 (2014).

15 Granja, J. M. *et al.* ArchR is a scalable software package for integrative single-cell chromatin accessibility analysis. *Nat Genet* **53**, 403-411, doi:10.1038/s41588-021-00790-6 (2021).

16 Ma, S. *et al.* Chromatin Potential Identified by Shared Single-Cell Profiling of RNA and Chromatin. *Cell* **183**, 1103-1116 e1120, doi:10.1016/j.cell.2020.09.056 (2020).

17 Qian, F.-C. *et al.* SEanalysis: a web tool for super-enhancer associated regulatory analysis. *Nucleic Acids Research* **47**, W248-W255, doi:10.1093/nar/gkz302 (2019).

18 Bravo Gonzalez-Blas, C. *et al.* cisTopic: cis-regulatory topic modeling on single-cell ATAC-seq data. *Nat Methods* **16**, 397-400, doi:10.1038/s41592-019-0367-1 (2019).
